# Gynecologic conditions in the context of incarceration: A scoping review

**DOI:** 10.1002/ijgo.70873

**Published:** 2026-02-27

**Authors:** Meredith K. Wise, Sahana Raghunathan, Sreya Upputuri, Elana Jaffe Brotkin, Tre D. Thorne, Jamie Conklin, Andrea K. Knittel

**Affiliations:** ^1^ Department of Obstetrics, Gynecology and Women's Health University of Minnesota Minneapolis Minnesota USA; ^2^ University of North Carolina at Chapel Hill School of Medicine Chapel Hill North Carolina USA; ^3^ University of North Carolina at Chapel Hill Chapel Hill North Carolina USA; ^4^ University of North Carolina at Chapel Hill Health Sciences Library Chapel Hill North Carolina USA; ^5^ Department of Obstetrics and Gynecology University of North Carolina at Chapel Hill Chapel Hill North Carolina USA

**Keywords:** gynecology, incarceration, jail, prison

## Abstract

**Background:**

More than 740 000 people identified as female at intake were incarcerated globally as of 2022, reflecting a 60% global increase since the year 2000, with a concomitant increase in gynecologic conditions experienced behind bars.

**Objectives:**

The purpose of this scoping review was to examine the breadth of benign and malignant gynecologic conditions experienced during incarceration, including the prevalence, special management considerations, access to services, and the patient experience.

**Method:**

The search strategy included a combination of keywords and subject headings for incarceration and benign or malignant gynecologic conditions with no language or date limits. Studies were eligible for the review if they: (i) discussed a benign or malignant gynecologic condition; (ii) included a population of people experiencing incarceration; and (iii) answered one or more of the four key questions identified prior to the search. One researcher independently screened each reference title and abstract for eligibility, and two reviewers independently screened each full text reference. One researcher extracted data from each study using a data extraction template, with verification and consensus by the primary and senior investigators.

**Results:**

After screening, 135 studies corresponding to 137 reports from 38 countries were included in the review. Included studies focused on cervical dysplasia and human papilloma virus (56), normal menstruation (38), vaginitis (36), routine gynecologic care (23), abnormal bleeding (17), pelvic pain (13), menopause (8), urinary incontinence (7), and gynecologic malignancy (5) during incarceration.

**Conclusion:**

The included studies demonstrate that across the globe, gynecologic conditions in carceral settings are common and can be exacerbated by the physical and emotional stress of incarceration, trauma histories, lack of access to care, and conditions of confinement in these settings. Gaps in the published literature exist on health education and interventions to address gynecologic health disparities and the gynecologic health needs of aging and older adults. There is a pressing need for parallel efforts at global de‐carceration and policy interventions to provide for basic gynecologic needs, decrease intersectional stigma, and improve the conditions of confinement.

## 1 INTRODUCTION

More than 740 000 people identified as female in intake are incarcerated globally as of 2022, reflecting a 60% global increase since the year 2000.^1^ The USA unfortunately leads this trend, with the increase in incarceration among people assigned female far outpacing both those assigned as male in the USA and female incarceration in all other countries.^1^, ^2^ The scope of this review is global, but considering national disparities in rates of incarceration, much of the framing centers on the US criminal legal system. In the USA, carceral settings include jails, which generally house people awaiting trial or serving short sentences, and prisons, which house people with longer sentences. The terminology used to describe facilities outside of the USA varies, as do the criminal legal structures that determine where and for how long individuals are incarcerated in those settings.^3^

Many gynecologic conditions, including the physiologic processes of menstruation and menopause, likely require accommodation for those experiencing incarceration in settings where people lack control over access to menstrual products or lifestyle modifications.^4^, ^5^ Moreover, managing common gynecologic concerns including abnormal uterine bleeding and cervical dysplasia, which might be more common or less well managed among people who are incarcerated, requires special considerations given the high prevalence of trauma history and the long‐documented history of reproductive coercion by carceral facilities and criminal legal system staff members.^5^, ^6^, ^7^, ^8^, ^9^

The American College of Obstetricians and Gynecologists states that people experiencing incarceration should have access to the full scope of reproductive care.^5^ Historically, however, research on the obstetric and gynecologic healthcare needs of people experiencing incarceration has focused on pregnancy care, sexually transmitted infections, and cervical dysplasia.^10^, ^11^, ^12^, ^13^ Recent reviews highlighting the difficult realities of contraception and abortion access in carceral settings and the ongoing high prevalence of human papilloma virus (HPV) and cervical dysplasia and cancer among people experiencing incarceration reflect the prioritization of these issues.^7^, ^14^, ^15^, ^16^

The aim of this review was to answer the following questions: (1) What is the prevalence of benign and malignant gynecologic conditions among people experiencing incarceration? (2) What are special considerations for managing benign and malignant gynecologic conditions in carceral settings? (3) What is access to services for benign and malignant gynecologic conditions like during incarceration? (4) How does incarceration affect experiences of benign and malignant gynecologic conditions?

## 2 METHODS

### 2.1 Reporting guidelines and registration

This review follows the Preferred Reporting Items for Systematic Reviews and Meta‐Analyses (PRISMA) extension for scoping reviews.^17^

### 2.2 Eligibility criteria

We considered carceral facilities to include jails, prisons, remand facilities, and other settings detaining people below the age of majority. Studies were eligible for the review if they (i) discussed a benign or malignant gynecologic condition; (ii) included a population of people experiencing incarceration; and (iii) answered one or more of the four key questions identified prior to the search. These questions were modeled after aspects of the Aday and Anderson Framework for the Study of Access to Care and addressed prevalence, special management considerations, access to services, and patient experience.^18^ All reference types were included except for review articles, commentaries, and book reviews. Gynecologic conditions included abnormal bleeding, menopause, pelvic pain, pelvic organ prolapse, urinary incontinence, cervical cancer screening, dysplasia, HPV, vaginitis, menstruation, and gynecologic malignancy. We also included general gynecologic care.

We excluded several groups of studies. First, studies focused on care for sexually transmitted infections, pregnancy, contraception, and abortion in carceral contexts have been covered extensively in previous reviews. Second, we excluded studies reviewed in two prior systematic reviews on the prevalence of cervical dysplasia and HPV in populations experiencing incarceration.^7^, ^16^ Third, we excluded studies conducted in World War II incarceration camps to avoid inclusion of data known to have been collected in violation of human rights. Studies describing US immigration detention centers were also excluded as they represent a part of the civil legal system.^1^, ^3^, ^19^ Finally, we excluded studies reporting on topics we did not consider benign or malignant gynecologic conditions: sexual behavior, forensics, sexual assault, domestic violence, intimate partner violence, and menstruation as a criminal defense or explanation of criminal behavior.

### 2.3 Information sources and search strategy

Our research team, including a health sciences librarian, developed the search strategy. With a final search date of April 19, 2023, the librarian searched APA PsycInfo (EBSCO*host*), CINAHL Plus with Full Text (EBSCO*host*), Embase (Elsevier), PubMed (https://pubmed.ncbi.nlm.nih.gov/), Scopus (Elsevier), and the ClinicalTrials.gov registry.

The search strategy included a combination of keywords and subject headings for incarceration and benign or malignant gynecologic conditions with no language or date limits. The PubMed search is shown in Table 1, and the complete, reproducible search strategy for all databases is available in the supplementary files. The research team also searched the cited references of all included studies.

**TABLE 1:** PubMed search strategy.

Set #	
1	“Correctional Facilities”[Mesh] OR “Prisoners”[Mesh] OR “Criminals”[Mesh] OR “Juvenile Delinquency”[Mesh] OR “mass incarceration”[tw] OR “incarcerated women”[tw] OR “incarcerated people”[tw] OR “incarcerated persons”[tw] OR “women incarcerated”[tw] OR “incarcerated youths”[tw] OR “incarcerated adolescent”[tw] OR “incarcerated adolescents”[tw] OR “adolescents incarcerated”[tw] OR “incarcerated girl”[tw] OR “incarcerated girls”[tw] OR “incarcerated patient”[tw] OR “incarcerated patients”[tw] OR “incarcerated individuals”[tw] OR “incarcerated populations”[tw] OR “incarcerated population”[tw] OR “incarcerated adults”[tw] OR “incarcerated adults”[tw] OR “incarcerated female”[tw] OR “incarcerated females”[tw] OR carceral[tw] OR “correctional facility”[tw] OR “correctional facilities”[tw] OR “correctional institution”[tw] OR “correctional institutions”[tw] OR “correctional center”[tw] OR “correctional centers”[tw] OR “correctional centre”[tw] OR “correctional centres”[tw] OR “correction facility”[tw] OR “correction facilities”[tw] OR “correction institution”[tw] OR “correction institutions”[tw] OR “correction center”[tw] OR “correction centers”[tw] OR “correction centre”[tw] OR “correction centres”[tw] “correctional health”[tw] OR “correctional healthcare”[tw] OR “correctional care”[tw] OR prisoner[tw] OR prisoners[tw] OR prison[tw] OR prisons[tw] OR imprisoned[tw] OR jail[tw] OR jails[tw] OR jailed[tw] OR inmate[tw] OR inmates[tw] OR criminal[tw] OR criminals[tw] OR felon[tw] OR felons[tw] OR offender[tw] OR offenders[tw] OR convict[tw] OR convicts[tw] OR “penal institution”[tw] OR “penal institutions”[tw] OR “penal facility”[tw] OR “penal facilities”[tw] OR penitentiary[tw] OR penitentiaries[tw] OR “justice involved”[tw] OR justice‐involved[tw] OR “detention centers”[tw] OR “detention center”[tw] OR “detention centres”[tw] OR “detention centre”[tw] OR “detention facility”[tw] OR “detention facilities”[tw] OR “juvenile detention”[tw] OR “youth detention”[tw] OR “juvenile delinquency”[tw] OR “juvenile justice”[tw]
2	“Gynecology”[Mesh] OR “Gynecological Examination”[Mesh] OR “Lower Urinary Tract Symptoms”[Mesh] OR “Papanicolaou Test”[Mesh] OR “Genital Diseases, Female”[Mesh] OR gynecology[tw] OR gynecology[tw] OR gynecologic[tw] OR gynaecologic[tw] OR gynecological[tw] OR gynecological[tw] OR gynecologist[tw] OR gynecologist[tw] OR gynecologists[tw] OR gynecologists[tw] OR urogynecologic[tw] OR urogynaecologic[tw] OR urogynecological[tw] OR urogynaecological[tw] OR urogynecology[tw] OR urogynaecology[tw] OR OBGYN[tw] OR OB‐GYN[tw] OR “female genital disease”[tw] OR “female genital diseases”[tw] OR “adnexal disease”[tw] OR “adnexal diseases”[tw] OR “adnexa disease”[tw] OR “adnexa diseases”[tw] OR “vaginal disease”[tw] OR “vaginal diseases”[tw] OR “vulvar disease”[tw] OR “vulvar diseases”[tw] OR “vulvovaginal disease”[tw] OR “vulvovaginal diseases”[tw] OR “vulvar diseases”[tw] OR “ovarian disease”[tw] OR “ovarian diseases”[tw] OR “ovarian cysts”[tw] OR “ovarian cyst”[tw] OR “Polycystic Ovary Syndrome”[tw] OR “uterine disease”[tw] OR “uterine diseases”[tw] OR “endometrial disease”[tw] OR “endometrial diseases”[tw] OR “cervical disease”[tw] OR “cervical diseases”[tw] OR vaginitis[tw] OR vulvovaginitis[tw] OR vulvitis[tw] OR vaginosis[tw] OR yeast[tw] OR candida[tw] OR Candidiasis[tw] OR “vaginal discharge”[tw] OR “vaginal discharges”[tw] OR “vaginal examination”[tw] OR “vaginal examinations”[tw] OR “vaginal exam”[tw] OR “vaginal exams”[tw] OR “pelvic examination”[tw] OR “pelvic examinations”[tw] OR “pelvic exam”[tw] OR “pelvic exams”[tw] OR Papanicolaou[tw] OR “pap test”[tw] OR “pap tests”[tw] OR “pap smear”[tw] OR “pap smears”[tw] OR “pap stain”[tw] OR “pap stains”[tw] OR “cervical smear”[tw] OR “cervical smears”[tw] OR “lower urinary tract symptoms”[tw]
3	“Uterine Hemorrhage”[Mesh] OR “uterine hemorrhage”[tw] OR “uterine hemorrhages”[tw] OR “uterine hemorrhaging”[tw] OR “uterus hemorrhage”[tw] OR “uterus hemorrhages”[tw] OR “uterus hemorrhaging”[tw] OR “uterine hemorrhage”[tw] OR “uterine hemorrhages”[tw] OR “uterine hemorrhaging”[tw] OR “uterus hemorrhage”[tw] OR “uterus hemorrhages”[tw] OR “uterus hemorrhaging”[tw] OR “uterine bleeding”[tw] OR “uterus bleeding”[tw] OR “vaginal hemorrhage”[tw] OR “vaginal hemorrhages”[tw] OR “vaginal hemorrhaging”[tw] OR “vaginal hemorrhage”[tw] OR “vaginal hemorrhages”[tw] OR “vaginal hemorrhaging”[tw] OR “vaginal bleeding”[tw] OR “vagina hemorrhage”[tw] OR “vagina hemorrhages”[tw] OR “vagina hemorrhaging”[tw] OR “vagina hemorrhage”[tw] OR “vagina hemorrhages”[tw] OR “vagina hemorrhaging”[tw] OR “vagina bleeding”[tw] OR “abnormal bleeding”[tw] OR “irregular bleeding”[tw] OR “abnormal menstrual bleeding”[tw] OR “abnormal menses”[tw] OR “Intermenstrual Bleeding”[tw] OR “Dysfunctional Uterine Bleeding”[tw] OR “Breakthrough Bleeding”[tw] OR “Bleeding Between Periods”[tw] OR “bleeding between menses”[tw] OR “heavy menstrual bleeding”[tw] OR “heavy menstruation”[tw] OR “heavy period”[tw] OR “heavy periods”[tw] OR “heavy menses”[tw] OR hypermenorrhea[tw] OR hypermenorrhoea[tw] OR menorrhagia[tw] OR metrorrhagia[tw] OR menometrorrhagia[tw] OR “irregular menstrual bleeding”[tw] OR “irregular menses”[tw]
4	“Menopause”[Mesh] OR menopaus*[tw] OR premenopaus*[tw] OR postmenopaus*[tw] OR perimenopause*[tw] OR “hot flashes”[tw] OR “hot flushes”[tw]
5	“Pelvic Pain”[Mesh] OR dysmenorrhea[tw] OR dysmenorrheas[tw] OR “piriformis muscle syndrome”[tw] OR “piriformis syndrome”[tw] OR endometriosis[tw] OR endometrioses[tw] OR ((“Pain”[Mesh] OR “Pain Management”[Mesh] OR pain*[tw]) AND (“Pelvis”[Mesh] OR pelvis[tw] OR pelvic[tw]))
6	“Genital Neoplasms, Female”[Mesh] OR “Squamous Intraepithelial Lesions of the Cervix”[tw] OR ((“Genitalia, Female”[Mesh] OR “female genital”[tw] OR “female genitals”[tw] OR “female genitalia”[tw] OR “female reproductive”[tw] OR Gynecologic[tw] OR cervical[tw] OR cervix[tw] OR endometrium[tw] OR endometria[tw] OR endometrial[tw] OR ovary[tw] OR ovaries[tw] OR ovarian[tw] OR uterus[tw] OR uteri[tw] OR womb[tw] OR wombs[tw] OR uterine[tw] OR vagina[tw] OR vaginas[tw] OR vaginal[tw] OR vulva[tw] OR vulvas[tw] OR vulvar[tw] OR “fallopian tube”[tw] OR “fallopian tubes”[tw]) AND (“Neoplasms”[Mesh] OR neoplasm[tw] OR neoplasms[tw] OR neoplasia[tw] OR cancer[tw] OR cancers[tw] OR cancerous[tw] OR carcinoma[tw] OR carcinomas[tw] OR malignant[tw] OR malignancy[tw] OR malignancies[tw] OR metastasis[tw] OR metastases[tw] OR metastatic[tw] OR tumor[tw] OR tumors[tw] OR tumor[tw] OR tumors[tw] OR sarcoma[tw] OR sarcomas[tw] OR oncology[tw] OR oncologist[tw] OR oncologists[tw] OR oncologica[tw]))
7	(“Pelvic Organ Prolapse”[Mesh] OR ((“Prolapse”[Mesh] OR prolapse[tw] OR prolapses[tw]) AND (“Pelvis”[Mesh] OR “Vagina”[Mesh] OR “Uterus”[Mesh] OR pelvis[tw] OR pelvic[tw] OR uterine[tw] OR visceral[tw] OR rectal[tw] OR vaginal[tw] OR vaginal[tw] OR urogenital[tw] OR genital[tw] OR urinary[tw] OR genitourinary[tw])))
8	(“Urinary Incontinence”[Mesh] OR “Nocturia”[Mesh] OR “Urinary Bladder, Overactive”[Mesh] OR “nocturnal diuresis”[tw] OR nocturia[tw] OR ((“Urination”[Mesh] OR “Urine”[Mesh] OR urinary[tw] OR urination[tw] OR micturition[tw] OR bladder[tw] OR urine[tw]) AND (incontinence[tw] OR incontinent[tw] OR urgency[tw] OR urge[tw] OR frequency[tw] OR leakage[tw] OR overactive[tw] OR overactivity[tw])))
9	(“Uterine Cervical Dysplasia”[Mesh] OR “Papillomavirus Infections”[Mesh] OR “Papillomaviridae”[Mesh] OR “abnormal pap”[tw] OR “abnormal paps”[tw] OR HPV[tw] OR “Human Papilloma Virus”[tw] OR “Human Papilloma Viruses”[tw] OR “Human Papillomavirus”[tw] OR “Papillomavirus Infection”[tw] OR “Papillomavirus Infections”[tw] OR “papillomaviral infection”[tw] OR “papillomaviral infections”[tw] OR ((Cervical[tw] OR cervix[tw] OR vaginal[tw] OR vagina[tw] OR vulvar[tw] OR vulva[tw] OR uterine[tw]) AND (dysplasia[tw] OR dysplasias[tw])))
10	(“Menstrual Cycle”[Mesh] OR “Menstruation”[Mesh] OR “Menstruation Disturbances”[Mesh] OR “Menarche”[Mesh] OR menstrual[tw] OR menstruation[tw] OR menstruating[tw] OR menarche[tw] OR menses[tw] OR premenstrual[tw] OR amenorrhea[tw])
11	#2 OR #3 OR #4 OR #5 OR #6 OR #7 OR #8 OR #9 OR #10
12	#1 AND #11

References were exported to Endnote X9 (Philadelphia, Pennsylvania, USA), where duplicates were removed. The remaining studies were imported into Covidence systematic review software (Veritas Health Innovation, Melbourne, Australia, available at www.covidence.org) to organize and complete the review process.

### 2.4 Study selection

One researcher independently screened each reference title and abstract for eligibility. In the full text review stage, two reviewers independently screened each reference. Conflicts were resolved by consensus of the full research team or by the lead author.

### 2.5 Data extraction and synthesis

We created a data extraction template capturing the reference title, lead author, year of publication, country, gynecologic condition, and key question type (prevalence, management, access, or experience). One researcher extracted data from each study, with a second reviewer verifying the extracted data.

We synthesized data according to the condition described and by the four key questions above pertaining to prevalence, management, access, or experience.

## 3 RESULTS

**FIGURE 1:**
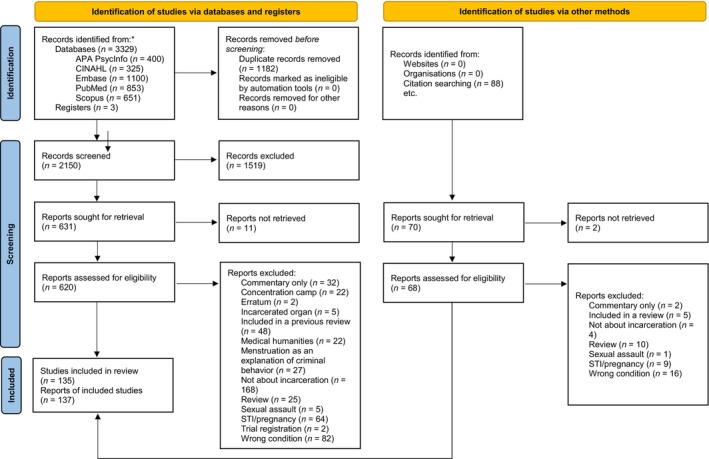
PRISMA flow diagram demonstrating study selection process.

We identified 3417 citations that met our search criteria (Figure 1). After removing duplicates, we assessed 2150 titles and abstracts and 688 full texts for eligibility; 135 studies were included, corresponding to 137 reports. Included studies addressed all four of the key questions, and most addressed more than one. The studies came from 38 distinct countries, with the majority from the USA (*n =* 49) Brazil (*n =* 17) and the UK (*n =* 13). There were a variety of study designs, with a majority being cross‐sectional descriptive studies, including many survey studies and chart review studies. There were also many studies that included qualitative methodologies. Some reports were published by governmental agencies and non‐governmental organizations. Because of the heterogeneity of data sources, the study design and methods were not always explicitly stated. The studies were conducted in a variety of settings, with a majority in prisons or jails, and a fair number in juvenile detention facilities.^6^, ^20^, ^21^, ^22^, ^23^, ^24^, ^25^, ^26^, ^27^, ^28^, ^29^, ^30^, ^31^, ^32^, ^33^, ^34^, ^35^, ^36^, ^37^, ^38^, ^39^, ^40^, ^41^, ^42^, ^43^, ^44^, ^45^, ^46^, ^47^, ^48^, ^49^, ^50^, ^51^, ^52^, ^53^, ^54^, ^55^, ^56^, ^57^, ^58^, ^59^, ^60^, ^61^, ^62^, ^63^, ^64^, ^65^, ^66^, ^67^, ^68^, ^69^, ^70^, ^71^, ^72^, ^73^, ^74^, ^75^, ^76^, ^77^, ^78^, ^79^, ^80^, ^81^, ^82^, ^83^, ^84^, ^85^, ^86^, ^87^, ^88^, ^89^, ^90^, ^91^, ^92^, ^93^, ^94^, ^95^, ^96^, ^97^, ^98^, ^99^, ^100^, ^101^, ^102^, ^103^, ^104^, ^105^, ^106^, ^107^, ^108^, ^109^, ^110^, ^111^, ^112^, ^113^, ^114^, ^115^, ^116^, ^117^, ^118^, ^119^, ^120^, ^121^, ^122^, ^123^, ^124^, ^125^, ^126^, ^127^, ^128^, ^129^, ^130^, ^131^, ^132^, ^133^, ^134^, ^135^, ^136^, ^137^, ^138^, ^139^, ^140^, ^141^, ^142^, ^143^, ^144^, ^145^, ^146^, ^147^, ^148^, ^149^, ^150^, ^151^, ^152^, ^153^, ^154^, ^155^

### 3.1 Gynecologic care and exams

Twenty‐three studies discussed the provision of routine or screening gynecologic care and exams, including 13 US studies, six from Brazil, two from the UK, and one each from Ghana and South Africa. Sixteen studies took place in prisons, three in jails, and four in other or a combination of facility types (Table 2).^46^, ^63^, ^65^, ^67^, ^69^, ^72^, ^76^, ^77^, ^79^, ^80^, ^81^, ^95^, ^97^, ^101^, ^107^, ^117^, ^118^, ^127^, ^136^, ^138^, ^145^, ^153^, ^155^ Two studies discussed the prevalence of requesting a gynecology appointment, and six studies discussed the prevalence of receiving gynecologic exams in carceral facilities.^46^, ^79^, ^80^, ^81^, ^97^, ^107^, ^117^, ^118^ A study in a Brazilian correctional facility for adolescents found that 13 of 26 (50%) individuals received a gynecological cancer prevention exam, with eight (61.5%) of those being more than 1 year ago.^97^ A study in a juvenile facility in the USA found that 87/133 (77%) of sexually active adolescents reported that they had a pelvic exam within the past year.^79^ Ingram‐Fogel found that in a prison in the USA, 72.7% of health care appointments made by participants were for gynecologic conditions, and 39.6% of the participants had “chronic” gynecological conditions.^81^ Several studies described rates of ongoing gynecologic care. Nicolau et al. found that 65 women (42%) received routine gynecologic care gynecologic care while incarcerated, ranging from every month to annually, and some were at inconsistent intervals.^117^, ^118^ De Araújo et al. found that in a Brazilian prison, 9% (*n =* 115) of women had never had a gynecological exam.^46^

**TABLE 2:** Studies addressing gynecologic care and exams.

Reference and year	Country	Setting	Prevalence	Special considerations for management	Access	Experiences
Centro pela Justiça e pelo Direito Internacional, 2007	Brazil	Prison			✓	
De Araújo et al., 2020	Brazil	Prison	✓		✓	
Fink et al., 1998	USA	Jail		✓		
Friedman et al., 2021	USA	Jail			✓	
Gallagher et al., 2007	USA	Other detention facility			✓	
Gender, Health & Justice Research Unit, 2012	South Africa	Prison			✓	✓
Grady, 1999	USA	Prison		✓	✓	✓
Harner and Burgess, 2011	USA	Prison		✓		✓
Hatton et al., 2006	USA	Jail			✓	✓
Holland‐Hall et al., 2002	USA	Other detention facility	✓	✓	✓	
Hyde et al., 2022	USA	Other detention facility	✓			
Ingram‐Fogel, 1991	USA	Prison	✓		✓	
Kraft‐Stolar, 2015	USA	Prison		✓	✓	✓
Leitie Araújo, 2019	Brazil	Other detention facility	✓			
Lowery et al., 2019	USA	Prison			✓	
Miranda et al., 2004	Brazil	Prison	✓			
Nicolau et al., 2012	Brazil	Prison	✓		✓	
Nicolau et al., 2015	Brazil	Prison	✓		✓	
Sabbagh Steinberg, 2018	USA	Prison		✓	✓	✓
Sarpong et al., 2015	Ghana	Prison		✓	✓	
Tang et al., 2010	UK	Prison			✓	
Woodall et al., 2021	UK	Prison			✓	
Young and Reviere, 2001	USA	Prison			✓	

Special considerations for routine gynecologic care discussed in seven studies included the need for gynecology‐focused educational initiatives and disparities in gynecologic care by socio‐demographic factors.^63^, ^72^, ^76^, ^79^, ^95^, ^136^, ^138^ In one of these, Fink et al. described a curriculum developed around gynecologic care in a correctional center instructing on four areas: anatomy and physiology, vaginal ecology, the pelvic exam, and sexual health.^63^ Gallagher et al. found that access varied in juvenile facilities and was significantly more likely in “all‐female, state‐owned, large populations and longer stay facilities” compared to short‐term, mixed gender, or locally‐ or privately‐operated facilities.^67^

Access to services was discussed in 18 studies, and key themes discussed were difficulty requesting appointments, lack of appropriate gynecologic healthcare, and financial burden of obtaining gynecologic care during incarceration.^46^, ^65^, ^67^, ^69^, ^72^, ^77^, ^79^, ^81^, ^95^, ^101^, ^117^, ^118^, ^127^, ^136^, ^138^, ^145^, ^153^, ^155^ Incarceration affecting experiences of gynecologic care and exams was discussed in five studies.^69^, ^72^, ^77^, ^95^, ^136^ For example, one US jail was reported to have inadequate health care for gynecologic conditions and unsanitary living conditions, such as residents being provided used underwear, causing them to develop gynecologic symptoms.^77^

Consistent and reliable access to gynecological care and exams is generally difficult in carceral settings and is complicated by the availability of gynecologic appointments and providers, patients' trust in their providers, and many of the social determinants of health that also affect care outside of carceral settings.

### 3.2 Normal menstruation

Thirty‐eight studies discussed normal menstruation during incarceration (Table 3).^29^, ^31^, ^35^, ^36^, ^39^, ^40^, ^41^, ^46^, ^51^, ^52^, ^64^, ^69^, ^71^, ^72^, ^73^, ^77^, ^82^, ^95^, ^97^, ^99^, ^102^, ^112^, ^114^, ^115^, ^120^, ^126^, ^127^, ^129^, ^134^, ^136^, ^137^, ^148^, ^149^, ^150^, ^151^, ^152^, ^155^, ^156^ The studies were performed in the USA (*n =* 10), the UK (*n =* 5), Brazil (*n =* 5), Zambia (*n =* 3), Cameroon (*n =* 2), Malawi (*n =* 2), and South Africa (*n =* 2), and one each in Ethiopia, French Guiana, Japan, Malawi, New Zealand, Nigeria, Somalia, and Slovakia, Uganda, and Zimbabwe. Twenty‐seven studies were conducted in prisons, two in jails, two in juvenile facilities, three in other detention facilities, and four in a combination of facility types. Twelve studies discussed the prevalence of normal menstruation, premenstrual syndrome, or details about menstruation such as age of menarche or length of menstrual cycle.^36^, ^46^, ^51^, ^52^, ^82^, ^97^, ^99^, ^112^, ^129^, ^134^, ^152^, ^156^ Three studies reported a prevalence of regular menstruation between 13/115 (11%) and 806/1198 (76.4%).^46^, ^82^, ^156^ In another, only one woman of 14 interviewed reported normal cycles.^52^ Several studies in juvenile facilities reported 12–13 years as the mean or modal age at menarche, similar to a comparison group from one study of non‐institutionalized young people.^36^, ^97^, ^99^, ^129^, ^152^ Studies reported a 29%–67% (20/29) prevalence of “PMS,” severe premenstrual symptoms, or bothersome periods.^36^, ^51^, ^112^, ^156^

**TABLE 3:** Studies addressing normal menstruation.

Reference and year	Country	Setting	Prevalence	Special considerations for management	Access	Experiences
Arua et al., 2023	Nigeria	Prison				
Bozelko et al., 2020	USA	Prison			✓	✓
Carlen, 1983	UK	Prison				✓
Carter, 1969	UK	Other detention facility	✓			
Centro pela Justiça e pelo Direito Internacional, 2007	Brazil	Prison			✓	
Chirwa, 2001	Uganda	Prison			✓	
Chirwa, 2002	Malawi	Prison, jail, ther detention facility			✓	
Chirwa, 2004	Ethiopia	Prison			✓	
de Araújo et al., 2020	Brazil	Prison	✓			
Deboscker et al., 2021	French Guiana	Prison	✓		✓	✓
de Sousa‐Rodrigues et al., 2006	Brazil	Prison	✓			
Fontebo, 2013	Cameroon	Prison			✓	✓
Gender, Health & Justice Research Unit, 2012	South Africa	Prison			✓	
Goodman et al., 2016	USA	Jail			✓	✓
Grady, 1999	USA	Prison			✓	
Gullberg, 2013	UK	Prison				✓
Hatton, 2006	USA	Jail			✓	
Ishihara, 1986	Japan	Juvenile facility	✓			
Kraft‐Stolar, 2015	USA	Prison			✓	✓
Leite Araújo et al., 2019	Brazil	Juvenile facility	✓			
Litt and Cohen, 1973	USA	Other detention facility	✓			
Luyt and Du Preez, 2010	South Africa	Prison, jail, other detention facility			✓	
Morton et al., 1953	USA	Prison, other detention facility	✓			
Nangia and Fontebo, 2017	Cameroon	Prison			✓	
Office of the Inspectorate Te Tari Tirohia	New Zealand	Prison			✓	✓
Plugge et al., 2006	UK	Prison			✓	
Ribeiro et al., 2013	Brazil	Prison	✓			
Rowe and Waters, 1935	USA	Prison	✓			
Sabbagh Steinberg, 2018	USA	Prison			✓	
Samakayi‐Makaratie, 2003	Zimbabwe	Prison			✓	✓
Smith, 2009	UK	Prison	✓			✓
Todrys and Amon, 2011	Zambia	Prison			✓	
Topp et al., 2016	Zambia	Prison			✓	
Topp et al., 2017	Zambia	Prison			✓	
Twea, 2004	Malawi	Prison			✓	
United Nations, 2012	Somalia	Prison, community alternatives			✓	
Valent et al., 1973	Slovakia	Other detention facility	✓			
Young and Reviere, 2001	USA	Prison			✓	

Twenty‐four studies discussed access to care for and management of normal menstruation, most of which centered on a lack of adequate supply of menstrual products, with many people relying on donations from outside groups like non‐governmental organizations or churches or on their families to bring in period management supplies.^30^, ^38^, ^39^, ^40^, ^51^, ^63^, ^68^, ^70^, ^71^, ^76^, ^94^, ^101^, ^113^, ^114^, ^119^, ^125^, ^126^, ^135^, ^136^, ^147^, ^148^, ^149^, ^150^, ^154^ The number of these studies is high, and they almost uniformly describe inadequate access to period products and a lack of autonomy in obtaining the products they need, with one study reporting that people must approach male correctional officers and provide specific reasons to request products.^72^ Another study discussed a lack of access to a toilet early in the morning, which was often when participants reported discovering that their period had started.^136^ One study, in contrast, stated that “83.89% [of respondents] indicated that the availability of women's hygiene articles was not a problem.”^102^ A survey found that 64/65 (98%) of institutions reported that they asked patients about menstrual cycles on intake and/or physical examinations.^155^ Outside discussions of access to management of normal menstruation and experiences of normal menstruation during incarceration, no studies focused on special considerations for management of normal menstruation in these settings.

Ten studies discussed experiences of normal menstruation by people experiencing incarceration.^31^, ^35^, ^52^, ^64^, ^71^, ^73^, ^95^, ^120^, ^137^, ^156^ Much of the focus of these was on the impact on self‐esteem and powerlessness that menstruating without proper access to period products has, how this is another area where the prison has control, and the people menstruating experience humiliation and violation of privacy.^31^, ^52^, ^71^, ^73^, ^95^, ^120^, ^156^ Studies also discussed the alternatives people used to manage menstruation in response to the lack of adequate supply of products, including using “newspapers by rubbing the numbers to make them more absorbent.”^137^

Many studies regarding access to products for managing normal menstruation and exploring the ways incarceration impacts experiences of normal menstruation met our inclusion criteria, and most focused on the inadequacy of period products and the shame and lack of privacy and autonomy that accompany menstruating while experiencing incarceration.

### 3.3 Abnormal uterine bleeding

Seventeen studies addressed abnormal uterine bleeding (AUB) (Table 4).^6^, ^21^, ^26^, ^44^, ^46^, ^52^, ^76^, ^77^, ^81^, ^82^, ^95^, ^126^, ^134^, ^140^, ^142^, ^144^, ^156^ These studies were performed in the USA (*n =* 8), Brazil (*n =* 2), the UK (*n =* 2), and one each in French Guiana, Greece, India, Italy, and Japan. They took place in prisons (*n =* 11), jails (*n =* 1), and other or multiple institutional facilities (*n =* 5). Prevalence of AUB was discussed in 13 studies and ranged from 6.4% (of 507) to 88.7% (*n =* 102).^6^, ^26^, ^44^, ^46^, ^52^, ^81^, ^82^, ^126^, ^134^, ^140^, ^142^, ^144^, ^156^ A study in a UK prison found that 49% of women reported their periods changed following imprisonment.^156^ Deboscker et al. found that nearly all study participants in a French Guianan prison experienced changes in their menstrual cycle during incarceration.^52^

**TABLE 4:** Studies addressing abnormal uterine bleeding.

Reference and year	Country	Setting	Prevalence	Special considerations for management	Access	Experiences
Adamopou‐lous et al., 1982	Greece	Other detention facility		✓		
Allsworth et al., 2007	USA	Prison and jail	✓			
Andrade et al., 2017	Brazil	Prison	✓			
D'Alessand‐ro et al., 1972	Italy	Other detention facility	✓			
de Araújo et al., 2020	Brazil	Prison	✓			
Deboscker et al., 2022	French Guiana	Prison	✓			
Harner and Burgess, 2011	USA	Prison		✓		
Hatton et al., 2006	USA	Jail				✓
Ingram‐Fogel, 1991	USA	Prison	✓		✓	
Ishihara, 1986	Japan	Juvenile facility	✓			
Kraft‐Stolar, 2015	USA	Prison		✓	✓	✓
Plugge et al., 2006	UK	Prison	✓	✓	✓	✓
Rowe and Waters, 2006	USA	Prison	✓			
Singla et al., 2020	India	Prison	✓			
Smith, 2009	UK	Prison	✓			✓
Stephens, 2015	USA	Juvenile facility	✓			
Sutcliffe et al., 2010	USA	Prison	✓			

Four studies emphasized special considerations for AUB, including a lack of access to hygiene products and trauma history.^21^, ^76^, ^95^, ^126^ Access to services was discussed in three studies, and key themes were the receipt of an inadequate number of menstrual products and a lack of full‐spectrum medical and surgical management options for AUB.^81^, ^95^, ^126^ For example, Kraft‐Stolar, in a New York state report, describes women reporting hysterectomies performed for abnormal uterine bleeding without proper education regarding alternatives to major surgery.^95^ In the USA, Ingram‐Fogel found that many individuals with menstrual difficulties reported not seeking health care as they found the prison system to be unresponsive, and they did not trust the health care they were given.^81^ The effect of incarceration on the experience of abnormal uterine bleeding was discussed in four studies.^77^, ^95^, ^126^, ^156^ Multiple studies discussed a lack of education for patients in carceral settings around the causes and options for management of abnormal uterine bleeding.

Overall, abnormal uterine bleeding is highly prevalent in carceral facilities, with patients reporting a lack of education around their conditions and diagnoses, decreased autonomy related to medical decision‐making, and not enough access to either menstrual products or gynecological providers while incarcerated.

### 3.4 Cervical dysplasia and human papillomavirus

After excluding studies included in past systematic reviews on cervical dysplasia and HPV in people experiencing incarceration, there were 56 studies that met our inclusion criteria (Table 5).^20^, ^23^, ^27^, ^28^, ^34^, ^38^, ^42^, ^43^, ^45^, ^46^, ^48^, ^49^, ^50^, ^53^, ^54^, ^55^, ^58^, ^59^, ^60^, ^62^, ^69^, ^78^, ^79^, ^81^, ^85^, ^86^, ^88^, ^89^, ^90^, ^91^, ^92^, ^94^, ^95^, ^96^, ^98^, ^104^, ^105^, ^106^, ^107^, ^108^, ^109^, ^111^, ^113^, ^118^, ^122^, ^123^, ^124^, ^125^, ^126^, ^129^, ^135^, ^140^, ^143^, ^146^, ^147^, ^155^ They were performed in the USA (*n =* 23), Brazil (*n =* 10), the UK (*n =* 3), Colombia (*n =* 2), India (*n =* 2), Russia (*n =* 2), Spain (*n =* 2), and one study each in Australia, Botswana, Canada, France, Ghana, Italy, Malta, Mexico, Peru, South Africa, Thailand, and Venezuela. Most took place in prisons (*n =* 37), and 10 were in jails, with 9 in other or multiple institutional facilities. Forty‐one studies reported a prevalence of cervical dysplasia (1/38 [2.6%]–47/157 [29.9%]) and HPV (8/214 [3.3%]–44/93 [52.7%]).^20^, ^23^, ^27^, ^28^, ^34^, ^38^, ^42^, ^43^, ^45^, ^48^, ^49^, ^50^, ^53^, ^54^, ^55^, ^60^, ^62^, ^79^, ^81^, ^85^, ^90^, ^91^, ^92^, ^96^, ^104^, ^105^, ^106^, ^107^, ^108^, ^109^, ^111^, ^113^, ^118^, ^123^, ^124^, ^125^, ^126^, ^129^, ^135^, ^140^, ^143^

**TABLE 5:** Studies addressing cervical dysplasia and HPV.

Reference and year	Country	Setting	Prevalence	Special considerations for management	Access	Experiences
Acheampong et al., 2021	Ghana	Prison	✓	✓		
Alcivar et al., 2020	Venezuela	Prison	✓			
Artymuk and Marochko, 2016	Russia	Prison	✓			
Artymuk and Marochko, 2016	Russia	Unspecified	✓			
Cañadas et al., 1998	Spain	Prison	✓			
Cerqueira et al., 1998	Brazil	Prison	✓			
Clarke et al., 2006	USA	Prison and jail	✓			
Coker et al., 1998	USA	Prison	✓			
D'Eça Junior et al., 2011	Brazil	Prison	✓			
De Araújo et al., 2020	Brazil	Prison			✓	
de Insuasty et al., 2008	Colombia	Prison	✓			
De Jesús Cabrera López et al., 2015	Mexico	Juvenile facility	✓			
De Sanjosé et al., 2000	Spain	Prison	✓			
Delage de Luget et al., 2022	France	Other detention facility	✓	✓		
Di Giussepe et al., 2022	Italy	Prison	✓	✓		
dos Anjos Sde et al., 2013	Brazil	Prison	✓			
Emerson et al., 2020	USA	Jail	✓	✓	✓	✓
Emerson et al., 2020	USA	Jail		✓		
Emerson et al., 2021	USA	Jail		✓	✓	
Farley et al., 2000	USA	Prison	✓			
Gender, Health & Justice Research Unit, 2012	South Africa	Prison			✓	
Henderson et al., 2010	USA	Other detention facility			✓	
Holland‐Hall et al., 2002	USA	Other detention facility	✓	✓	✓	
Ingram‐Fogel, 1991	USA	Prison	✓		✓	
Jiamset et al., 2019	Thailand	Jail	✓		✓	
Kanbergs et al., 2023	USA	Prison		✓	✓	
Kelly et al., 2017	USA	Jail		✓		✓
Kelly et al., 2022	USA	Jail		✓		
Kesha et al., 2011	USA	Prison	✓			
Kim et al., 2006	USA	Jail	✓	✓		
Kim et al., 2011	USA	Jail	✓			
Kouyoumdjian et al., 2018	Canada	Prison and jail			✓	
Kraft‐Stolar, 2015	USA	Prison		✓	✓	✓
Lee, 1987	Australia	Other detention facility	✓			
Lipnicky et al., 2022	USA	Jail			✓	
Mahto and Zia, 2008	UK	Prison	✓		✓	
Mehta et al., 2020	India	Prison	✓			
Michelsen, 1969	Colombia	Prison	✓			
Miranda et al., 2000	Brazil	Prison	✓			
Miranda et al., 2004	Brazil	Prison	✓			
Modie‐Moroka, 2003	Botswana	Prison	✓			
Moore et al., 2019	USA	Prison	✓		✓	✓
Muscat et al., 2022	Malta	Prison	✓			
Nicolau et al., 2012	Brazil	Prison	✓		✓	
Pereira Borges et al., 2018	Brazil	Prison			✓	
Pereyra, 1961	USA	Prison	✓			
Philipp, 1987	UK	Prison	✓			
Pickett et al., 2018	USA	Jail	✓			
Plugge et al., 2006	UK	Prison	✓		✓	
Ribeiro et al., 2013	Brazil	Prison	✓			
Ruiz‐Maza et al., 2018	Peru	Prison	✓	✓	✓	
Singla and Mehta, 2022	India	Prison	✓			
Stevens and Zierler, 1995	USA	Prison	✓			
Tavares de Oliveira et al., 2020	Brazil	Prison			✓	
Teck et al., 2016	USA	Other detention facility		✓		
Young and Reviere, 2001	USA	Prison			✓	

Special considerations affecting management of HPV and cervical dysplasia in people experiencing incarceration were discussed in 14 studies and often centered on HPV vaccination status and histories of sexual abuse.^20^, ^53^, ^54^, ^58^, ^59^, ^60^, ^79^, ^86^, ^88^, ^89^, ^92^, ^95^, ^135^, ^147^ Other key themes included a delay or lack of proper follow‐up care or procedures, structural and financial challenges, competing demands, and lack of stability.^89^, ^95^ A study in a US prison found that women who had sexual intercourse at an earlier age or experienced sexual abuse were more likely to enter prison with cervical dysplasia.^43^ Emerson et al. found that in a US jail, age was a risk factor for out‐of‐date Pap smears; the odds of up‐to‐date cervical cancer screening decreased by 5% (*P* < 0.05) for every 1‐year increase in age.^60^

Access to services was discussed in 20 studies.^46^, ^59^, ^60^, ^69^, ^78^, ^79^, ^81^, ^85^, ^86^, ^94^, ^95^, ^98^, ^104^, ^111^, ^118^, ^122^, ^126^, ^135^, ^146^ Pereira Borges reported that 38 of 56 women (67.86%) incarcerated in a Brazilian prison received guideline‐concordant screening.^122^ US carceral facilities and administrators who believed offering the HPV vaccine was important were more likely to provide other preventive sexual health services; just 39 of 50 states reported offering the HPV vaccine to incarcerated females in juvenile facilities.^78^, ^98^ Incarceration affecting experiences of cervical dysplasia and HPV was discussed in four studies.^60^, ^89^, ^95^, ^111^

Women with a history of incarceration had higher rates of reported cervical cancer and abnormal Pap smears compared to women without this history.^58^ There is an abundance of data on cervical dysplasia and HPV compared to the other gynecological conditions studied, detailing the high burden of these conditions in populations experiencing incarceration and the challenges of achieving universal preventive, screening, and follow‐up care.

### 3.5 Genital warts

Eleven studies addressed genital warts in prisons (*n =* 2), juvenile facilities (*n =* 3), other detention facilities (*n =* 3) or a combination of settings (*n =* 3) (Table 6).^25^, ^32^, ^42^, ^49^, ^54^, ^62^, ^66^, ^104^, ^119^, ^133^, ^142^ Five studies were performed in the USA, three in the UK, and one each in Australia, Italy, and Mexico. From 10 studies, the prevalence of genital warts was 3/110 (2.7%)–12/132 (9.1%), 36/378 (9.5%) while incarcerated and 33/474 (6.9%) of participants reporting a history of prior genital warts. Examining conditions with shared risk factors, Nijhawan et al. reported an equal rate of genital warts in patients who tested positive for trichomonas (*n =* 5/53, 10%) and those who did not (*n =* 31/325, 10%), and a study of juveniles experiencing incarceration reported that 1.7% of adolescents disclosing ecstasy use reported genital warts in the past year compared to 1.6% among those without ecstasy use (OR 0.91, 95% CI 0.32–2.61).^119^, ^142^

**TABLE 6:** Studies addressing genital warts.

Reference and year	Country	Setting	Prevalence	Special considerations for management	Access	Experiences
Alward, 1966	USA	Other detention facility	✓			
Butler et al., 2000	Australia	Prison, other detention facility	✓			
Clarke et al., 2006	USA	Prison and jail	✓			
De Jesús Cabrera López et al., 2015	Mexico	Juvenile facility	✓			
Di Giuseppe et al., 2022	Italy	Prison		✓		✓
Farley et al., 2000	USA	Prison	✓			
Gallagher, 1970	UK	Other detention facility	✓			
Mahto and Zia, 2008	UK	Prison	✓		✓	
Nijhawan et al., 2012	USA	Prison and jail	✓			
Robertson and George, 1970	UK	Juvenile facility	✓			
Stephens et al., 2015	USA	Juvenile facility	✓			

One study discussed special considerations for managing genital warts in carceral settings, noting that only 84/214 (39.2%) of participants in a survey knew that HPV vaccination could prevent genital warts.^54^ One study discussed access to services for patients with genital warts, noting a new female genito‐urinary consultant was hired to increase onsite testing and access to treatment for conditions including genital warts.^104^ One study discussed experiences of genital warts in the carceral setting, with 152 of 214 (71%) juvenile facility participants reporting that they would feel embarrassed to have genital warts.^54^ Prevalence of genital warts was reported in many studies that met our inclusion criteria, but there were limited data addressing the other key questions.

### 3.6 Gynecologic malignancies

There were five studies (USA, *n =* 3, UK, *n =* 2) that discussed gynecologic malignancies among people experiencing incarceration; four were conducted in prisons and one in a combination of prison and jail settings (Table 7).^30^, ^87^, ^95^, ^123^, ^124^ Prevalence of gynecologic malignancies was discussed in four studies, all of which reported on the prevalence of cervical cancer. The prevalence of cervical cancer ranged from 6/583 (1.0%) to 15/601 (2.5%).^87^, ^123^ Another study reported a 9% combined prevalence of premalignant or malignant conditions of the cervix.^124^ In the fourth, individuals in jails and prisons had increased odds of cervical cancer compared to a non‐institutionalized population (OR for jail 4.16, 95% CI 3.13–5.53, OR for prison, 4.82, 95% CI 3.74–6.22).^30^ One study also mentioned that “several cases of ovarian cancer were also diagnosed” but did not report a specific prevalence.^124^ Access to services for gynecologic malignancies was discussed in one study, which stated that “patients reported delays in gyn care leading to symptoms worsening and delays in diagnosis and treatment of cancer and cervical dysplasia.”^95^

**TABLE 7:** Studies addressing gynecologic malignancies.

Reference and year	Country	Setting	Prevalence	Special considerations for management	Access	Experiences
Binswanger et al., 2009	USA	Prison and jail	✓			
Keighley, 1871	UK	Prison	✓			
Kraft‐Stolar, 2015	USA	Prison			✓	
Pereyra, 1961	USA	Prison	✓			
Philipp, 1987	UK	Prison	✓			

Special considerations for management of gynecologic malignancies for people experiencing incarceration and experiences of gynecologic malignancies in carceral settings were not discussed in any studies. There are minimal data on gynecologic malignancies in populations experiencing incarceration, and what does exist largely focuses on cervical cancer, further highlighting both the burden of and emphasis on cervical dysplasia and cancer that occurs in this population.

### 3.7 Menopause

There were eight studies that discussed menopause during incarceration (Table 8).^22^, ^47^, ^72^, ^83^, ^84^, ^139^, ^153^, ^155^ Five studies were performed in the USA, one in the UK, one in Brazil, and one in Germany. Six studies took place in prisons and one in both prison and jail; the German study was conducted in an unspecified facility. Two studies reported on prevalence: a study in southeastern US prisons found that 30% of 327 of women surveyed reported “menopause problems,” with a higher rate in Black women compared to non‐Black women.^22^ In a North Carolina prison, 382/863 (32.8%) of all prescriptions in the facility were found to be relevant to menopause; the most prescribed medication type that can be used to manage menopause (*n =* 346, 30.9%) was selective serotonin reuptake inhibitors, and 68 (6.1%) were hormonal therapies.^84^ Special considerations affecting menopause care during incarceration, discussed in five studies, included the impact of coexisting medical conditions and poor general health.^22^, ^72^, ^83^, ^84^, ^139^ Two studies discussed the high prevalence of coexisting medical conditions, including hypertension, cardiovascular conditions, and mental health conditions in people experiencing incarceration that complicates management of^22^, ^84^ Jaffe et al. reported a rate of 40/1120 (3.6%) of individuals aged 45–75 receiving estrogen‐containing menopausal hormone therapy.^84^

**TABLE 8:** Studies addressing menopause.

Reference and year	Country	Setting	Prevalence	Special considerations for management	Access	Experiences
Aday and Farney, 2014	USA	Prison	✓	✓		
de Cássia Ferreira et al., 2017	Brazil	Prison				✓
Grady, 1999	USA	Prison		✓		
Jaffe et al., 2021	USA	Jail and Prison		✓	✓	✓
Jaffe et al., 2022	USA	Prison	✓	✓		
Schneider‐Reinkens, 1969	Germany	Other Detention Facility		✓		
Woodall et al., 2021	UK	Prison			✓	
Young and Reveire, 2001	USA	Prison			✓	

Access to services was discussed in three studies.^83^, ^153^, ^155^ Two studies only briefly mentioned that the patients in the facilities they studied were asked about symptoms of menopause or provided education about menopause.^153^, ^155^ One qualitative study discussed a lack of access to lifestyle management of menopause, including replacement clothing and clean bedding to manage abnormal bleeding and night sweats associated with the menopause transition as well as few ceiling fans and no air conditioning.^83^ Incarceration affecting the experience of menopause was discussed in two studies.^47^, ^83^ Participants raised issues including a lack of menopause knowledge and the threat of disciplinary action for certain methods of coping with menopause symptoms such as sleeping without underwear to help with hot flushes.^83^

The studies on menopause in carceral settings highlight the complexity of caring for these patients given their lack of access to lifestyle management techniques and the high prevalence of comorbid conditions that affect pharmacologic management of menopause.

### 3.8 Pelvic organ prolapse

There were no studies that discussed pelvic organ prolapse in individuals experiencing incarceration.

### 3.9 Pelvic pain

Thirteen studies discussed pelvic pain, although this was not the primary focus of any of these studies, but rather the discussion of pelvic pain was embedded in studies on other topics, most commonly in studies on sexually transmitted infections (Table 9).^46^, ^70^, ^74^, ^77^, ^95^, ^108^, ^109^, ^128^, ^140^, ^142^, ^144^, ^154^, ^156^ These studies were from the USA (*n =* 5), Brazil (*n =* 2), and one each in Botswana, Ethiopia, India, Switzerland, and the UK; 10 were conducted in prisons, one in a jail, and two in juvenile facilities. Twelve of these studies reported the prevalence of pelvic pain in patients experiencing incarceration, which ranged from 5.1% (*n =* 6/121) to 54% (of 111).^46^, ^70^, ^74^, ^105^, ^107^, ^108^, ^109^, ^128^, ^142^, ^144^, ^154^, ^156^ Most studies reported on pelvic pain in general or primary dysmenorrhea, although the focus of one paper was on bladder pain, and another reported the prevalence of “endometriosis/ovarian cysts.”^128^, ^154^

**TABLE 9:** Studies addressing pelvic pain.

Reference and year	Country	Setting	Prevalence	Special considerations for management	Access	Experiences
de Araújo et al., 2020	Brazil	Prison	✓			
Getachew et al., 2021	Ethiopia	Prison	✓			
Haller et al., 2010	Switzerland	Juvenile facility	✓			
Hatton et al., 2006	USA	Jail			✓	✓
Kraft‐Stolar, 2015	USA	Prison		✓		✓
Miranda et al., 2000	Brazil	Prison	✓			
Modie‐Moroka, 2003	Botswana	Prison	✓			✓
Ribeiro De Menenzes et al., 2021	Brazil	Prison	✓			✓
Singla et al., 2020	India	Prison	✓			
Smith, 2009	UK	Prison	✓			✓
Sutcliffe et al., 2010	USA	Prison	✓			
Stephens et al., 2015	USA	Juvenile facility	✓			
Young, 1998	USA	Prison	✓			

One study discussed special considerations for management of pelvic pain, reviewing the importance of informed consent for treatment of patients experiencing incarceration.^95^ One study mentioned access to services for pelvic pain with study participants finding “health care inappropriate for their needs and not set up to handle female diseases … [including] endometriosis.”^77^ Five studies discussed experiences of pelvic pain while incarcerated.^77^, ^95^, ^109^, ^128^, ^156^ A participant in one study reported feeling disempowered and distressed by the care she received related to pelvic pain due to a lack of adequate explanation.^95^ In another study, participants reported that their dysmenorrhea worsened during their incarceration.^156^

These studies highlight the range of prevalence of pelvic pain in people experiencing incarceration. The qualitative studies that include direct quotes from participants highlight the impact incarceration has on pelvic symptoms, contributing to worsening pain and a lack of control in managing these symptoms.

### 3.10 Urinary incontinence

Seven studies addressed urinary incontinence, including two from the USA, two from the UK, and one each from Australia, Brazil, and Pakistan (Table 10).^56^, ^72^, ^103^, ^124^, ^128^, ^130^, ^155^ Six studies took place in prisons, and one took place in a jail. Five of these discussed the prevalence of urinary incontinence, ranging from 14% (2/14) to 93%, with one study reporting that most patients over the age of 50 have urinary incontinence.^56^, ^103^, ^124^, ^128^, ^130^ One study discussed special considerations for managing urinary incontinence in the carceral setting, citing one patient's inability to follow recommendations to keep a voiding diary due to the diary being confiscated by an officer.^72^ Three studies discussed access to services for patients with urinary incontinence.^56^, ^72^, ^155^ One survey found that 46/65 (71%) of respondents reported being asked about urinary incontinence at their intake visit.^155^ Another mentions that patients must ask officers for incontinence pads.^72^

**TABLE 10:** Studies addressing urinary incontinence.

Reference and year	Country	Setting	Prevalence	Special considerations for management	Access	Experiences
Drennan et al., 2010	UK	Prison	✓		✓	✓
Grady, 1999	USA	Prison		✓	✓	✓
Mahmood et al., 2020	Pakistan	Jail	✓			
Ribeiro de Menezes, 2021	Brazil	Prison	✓			✓
Rice et al., 2021	Australia	Prison	✓			
Philipp, 1987	UK	Prison	✓			
Young and Reviere, 2001	USA	Prison			✓	

Three studies discussed experiences of urinary incontinence during incarceration, highlighting the condition's impact on quality of life. Reported experiences included restricting food and water intake to manage incontinence, worrying about malodor due to incontinence, and choosing to delay treatment until a return to the community.^56^, ^72^, ^128^ The studies demonstrate a relatively high prevalence of urinary incontinence in people experiencing incarceration and the barriers that providers and patients experience in managing urinary incontinence during incarceration, including lack of privacy and lack of free access to sanitary supplies.

### 3.11 Vaginitis

There were 36 studies that discussed vaginitis among people experiencing incarceration (Table 11).^24^, ^33^, ^37^, ^61^, ^62^, ^65^, ^66^, ^68^, ^70^, ^74^, ^75^, ^77^, ^79^, ^87^, ^93^, ^95^, ^96^, ^97^, ^100^, ^104^, ^107^, ^108^, ^113^, ^116^, ^119^, ^121^, ^122^, ^129^, ^131^, ^132^, ^136^, ^138^, ^142^, ^144^, ^152^, ^154^ The studies were conducted in the USA (*n =* 11), Brazil (*n =* 5), the UK (*n =* 3), two each in Australia, Slovakia, Spain, and Switzerland, and one each in Canada, Chile, Colombia, Ethiopia, Ghana, Iran, Portugal, and Malta; 20 were conducted in prisons, three in jails, one in a combination of prison and jail sites, eight in juvenile facilities, and four in other detention facilities. Prevalence of vaginitis was defined variably across 32 studies as the presence of specific microorganisms, vaginal discharge, or vaginal pruritis.^24^, ^33^, ^37^, ^61^, ^62^, ^66^, ^68^, ^70^, ^74^, ^75^, ^79^, ^87^, ^93^, ^96^, ^97^, ^100^, ^104^, ^107^, ^108^, ^113^, ^116^, ^119^, ^121^, ^122^, ^129^, ^131^, ^132^, ^136^, ^142^, ^144^, ^152^, ^154^

**TABLE 11:** Studies addressing vaginitis.

Reference and year	Country	Setting	Prevalence	Special considerations for management	Access	Experiences
Alonso‐Sanz et al., 1996	Spain	Prison	✓			
Caloenescu et al., 1973	Canada	Other detention facility	✓			
Celia Bórquez et al., 2022	Chile	Prison	✓			
Farhoudi et al., 2022	Iran	Prison	✓			
Farley et al., 2000	USA	Prison	✓			
Friedman et al., 2021	USA	Jail			✓	
Gallagher, 1970	UK	Other detention facility	✓			
Garcia et al., 2004	Portugal	Prison	✓			
Getachew et al., 2021	Ethiopia	Prison	✓			
Haller et al., 2009	Switzerland	Juvenile facility	✓			
Haller et al., 2010	Switzerland	Juvenile facility	✓			
Hatton et al., 2006	USA	Jail			✓	✓
Holland‐Hall et al., 2002	USA	Other detention facility	✓	✓	✓	
Keighley, 1971	UK	Prison	✓			
Klobusický et al., 1989	Slovakia	Prison	✓			
Kraft‐Stolar, 2006	USA	Prison				✓
Lee, 1987	Australia	Jevenile facility	✓			
Leite Araújo et al., 2019	Brazil	Juvenile facility	✓			
López‐Barbosa et al., 2009	Colombia	Jail	✓			
Mahto and Zia, 2008	UK	Prison	✓		✓	
Miranda et al., 2000	Brazil	Prison	✓			
Miranda et al., 2004	Brazil	Prison	✓			
Muscat et al., 2022	Malta	Prison	✓			
Nicholson et al., 2003	Australia	Prison	✓			
Nijhawan et al., 2012	USA	Jail and Prison	✓			
Oliván Gonzalvo, 2002	Spain	Other detention facility	✓			
Pereira Borges et al., 2018	Brazil	Prison	✓			
Ribeiro et al., 2013	Brazil	Prison	✓			
Ris and Dodge, 1973	USA	Juvenile facility	✓			
Risser et al., 2004	USA	Juvenile facility	✓			
Sabbagh Steinberg, 2018	USA	Prison	✓	✓	✓	
Sarpong et al., 2015	Ghana	Prison		✓	✓	
Stephens et al., 2015	USA	Juvenile facility	✓			
Sutcliffe et al., 2010	USA	Prison	✓			
Valent et al., 1973	Slovakia	Juvenile facility	✓			
Young, 1998	USA	Prison	✓			

Overall vaginitis rates ranged from *n =* 5/85 (6%) to *n =* 16/26 (62%).^24^, ^61^, ^70^, ^74^, ^75^, ^97^, ^107^, ^108^, ^119^, ^122^, ^129^, ^144^, ^154^ Several studies reported specific rates of *Gardnerella vaginalis* or bacterial vaginosis (7/66 [3%]–10/25 [40%]), trichomonas vaginalis (25/583 [4.3%]–329/556 [59%]), and candida species or yeast organisms (1/53 [2%]–91/486 [19%]).^24^, ^33^, ^37^, ^68^, ^74^, ^79^, ^93^, ^96^, ^100^, ^104^, ^108^, ^113^, ^116^, ^119^, ^121^, ^131^, ^144^, ^152^ Among women with HIV incarcerated in Rhode Island, 27/110 (25%) had vaginal candidiasis.^62^ Among patients being treated for trichomoniasis, there was a co‐infection rate of 25/583 (4%) for candida vaginitis.^87^ Individuals in a juvenile facility who had used ecstasy previously were more likely to report vaginal discharge or odor (OR 2.33, 95% CI 1.16–4.65).^142^

Special considerations for management of vaginitis in carceral settings were discussed in two studies and included giving patients the option to self‐test and the high rate of recurrence attributed to limited hygiene facilities.^79^, ^138^ Access to services was discussed in four studies.^65^, ^77^, ^104^, ^138^ One study described the process of accessing care and included a quote from a patient who had received advice from a nurse to exaggerate symptoms to have requests for medical care related to vaginitis be taken seriously.^65^ Another study found that women identified a lack of resources to maintain hygiene as a contributor to the development of vaginitis.^77^ One study improved access for vaginitis care by hiring a female genitourinary consultant.^104^ A study from Ghana described an intermittent lack of availability of drugs to treat candidiasis.^138^

Incarceration affecting experiences of vaginitis was discussed in one study that mentioned a patient feeling like they were not receiving adequate communication or education as to why they were having recurrent yeast infections.

Prevalence of vaginitis was commonly reported in studies about gynecologic care in carceral settings, highlighting the commonality of this complaint in this population. Experiences of vaginitis in carceral settings centered on difficulty accessing care for treatment and theorizing that hygiene practices in some facilities affected vaginitis rates.

### 3.12 Other gynecologic conditions

Four studies addressed the prevalence of other conditions (Table 12).^25^, ^26^, ^134^, ^154^ Young reported a rate of 6/129 (4.7%) of “endometriosis/ovarian cysts” in a US prison.^154^ Rowe and Waters noted premature ovarian failure in 25 patients in a Massachusetts facility in 1935 (one attributed to oophorectomy, 10 attributed to prior gonorrhea infection, and 10 without a proposed etiology).^134^ A 1965 presentation of data from a Los Angeles juvenile facility reported 102 cases of cervical erosion and one case of an absent vagina.^25^ Andrade et al. reported a rate of 58/146 (39.72%) of ultrasound abnormalities in 146 patients experiencing menstrual irregularities in a Brazil prison.^26^

**TABLE 12:** Studies addressing other conditions.

Reference and year	Country	Setting	Condition	Prevalence	Special considerations for management	Access	Experiences
Alward, 1966	USA	Juvenile facility	Erosion of cervix, Müllerian anomalies	✓			
Andrade, et al., 2017	Brazil	Prison	Pelvic ultrasound abnromalities	✓			
Rowe and Waters, 1935	USA	Prison	Ovarian failure	✓			
Young, 1998	USA	Prison	Ovarian cysts	✓			

## 4 DISCUSSION

Synthesis of the 135 studies included in this review demonstrated both that gynecologic conditions are common in carceral settings and that disparities in the experience of gynecologic conditions have three underpinning mechanisms. First, there are gynecologic conditions that are brought on or exacerbated by the physical and emotional stress of incarceration, such as abnormal uterine bleeding, pelvic pain, or menopausal symptoms. Second, there are shared social and structural factors, such as substance use and physical, emotional, and sexual abuse and trauma that are common among individuals experiencing incarceration and might exacerbate some gynecologic disparities (e.g., cervical dysplasia and cancer). Third, conditions of confinement, lack of access to care, and intersectional stigma intensify the physical and emotional experience of some conditions, including normal menstruation, urinary incontinence, and pelvic pain.

These findings enrich and extend the findings of previous, condition‐specific reviews. Conducting this literature review across multiple gynecologic conditions, languages, global contexts, and many decades (1930s‐present) resulted in several important throughlines. A unifying theme across time and place was the lack of adequate accommodations and supplies to manage normal menstruation or any gynecologic conditions that a person might experience during incarceration. Additionally, although we excluded studies focused exclusively on sexually transmitted infections as well as the many studies of cervical dysplasia included in recent systematic reviews, the most frequently addressed gynecologic conditions in our review, apart from normal menstruation, were cervical dysplasia and vaginitis. The persistent and international emphasis on gynecologic conditions related to sexual health would seem to belie the other gynecologic health disparities experienced by people who are incarcerated. Finally, although there have been more descriptions of interventions to improve care for gynecologic health conditions in carceral settings in recent years, there are relatively few studies proposing or testing interventions to address these health needs compared with the number of descriptive studies.

There are important policy implications from our findings. Although mass de‐carceration is likely the most straightforward approach to minimizing the gynecologic disparities that result from incarceration, interim steps to improve conditions of confinement are needed. The most urgent of these might be the provision of adequate sanitary supplies for people experiencing normal menstruation, abnormal uterine bleeding, and urinary incontinence. Although philosophies of incarceration and punishment vary across settings, the United Nations Basic Principles for Treatment of Prisoners require treatment “with respect due to their inherent dignity and value as human beings,” precluding shaming and/or degrading incarcerated individuals for their bodily functions.^157^

Adequate provision of basic supplies will go a long way toward the humane management of gynecologic conditions, and studies on normal and abnormal menstruation, pelvic pain, urinary incontinence, and vaginitis also emphasized the importance of decreasing stigma and promoting bodily autonomy and self‐determination. The practice of trauma‐informed care (i.e., care that identifies trauma and related symptoms, trains staff on the effects of trauma, and minimizes re‐traumatization) offers a way forward in clinical spaces.^158^ Programs such as Amend at the University of California, San Francisco, which bring dignity‐driven and public health‐oriented practices from Norway and elsewhere to improve the culture in US prisons, can serve as a roadmap for carceral systems where trauma‐informed care is not yet the norm.^159^

There are also critical changes needed globally to the systems of care that people access during incarceration. The studies we reviewed evinced substantial concerns expressed about the lack of competent and trustworthy providers in the context of accessing routine gynecologic care and care for abnormal uterine bleeding, menopause, and vaginitis. Addressing these gaps in the provider workforce will require multi‐pronged efforts to dismiss or remediate providers who are not prepared to provide trauma‐informed, community‐standard care and to make clinical positions within carceral facilities desirable to providers with the necessary skills. Important steps to accomplish the latter will include improving the physical and human resources in carceral clinical settings, actively destigmatizing carceral medicine as a valued public service career path and establishing universal training in trauma‐informed care.

Beyond improvements to clinical services, studies across multiple gynecologic conditions also identified a need for health education about abnormal uterine bleeding and menopause. Although there have been reports of educational interventions focused on reducing risk for STIs including HIV, similar educational programs focused on non‐sexually transmitted gynecologic conditions are limited.^160^, ^161^ In addition, although the authors are aware of educational activities outside the USA (Knittel, personal communication), fewer of these interventions have been published in the peer‐reviewed literature. Taking inspiration from the internet‐based sexual health empowerment curriculum developed in Midwestern USA, health researchers and educators globally would do well to create low‐cost, easily disseminated, and scientifically accurate educational resources about gynecologic conditions across the life course.^162^

Finally, this synthesis of global literature reveals a substantial gap in the literature on studies of conditions that disproportionately affect aging and older adults. We found relatively few studies examining either gynecologic cancer experiences and outcomes or menopause experiences and no studies addressing pelvic organ prolapse. This gap remains more than a decade after the publication of a policy setting agenda to address disparities for those aging in custody that included recognition of their gynecologic needs.^163^

This review is not without limitations. Our scoping review methodology included a wide range of studies that varied in terms of methods, clinical focus, and quality. While this allowed us to review the global landscape of gynecologic conditions in settings of incarceration, the breadth precluded meta‐analysis or other quantitative approaches to synthesize findings. We were also limited in our review by a relative lack of intervention trials and publications describing ways to address the gynecologic health disparities identified.

## 5 CONCLUSION

In summary, in carceral settings across the globe, gynecologic conditions are common and often exacerbated by the physical and emotional stress of incarceration, trauma histories, lack of access to care, and conditions of confinement in these settings. Additional research efforts are needed in the arenas of health education and interventions to address gynecologic health disparities, as well as to increase understanding of the gynecologic health needs of aging and older adults. Most pressing, however, is the need for parallel efforts at global de‐carceration and policy interventions to provide for basic gynecologic needs, decrease intersectional stigma, and improve the conditions of confinement.

## AUTHOR CONTRIBUTIONS

Meredith K. Wise: review conceptualization, search strategy design, abstract and full text review, data abstraction, data synthesis, manuscript drafting, final manuscript approval; Sahana Raghunathan: data abstraction, data synthesis, manuscript drafting, final manuscript approval; Sreya Upputuri: abstract and full text review, data abstraction, final manuscript approval; Elana Jaffe Brotkin: abstract and full text review, data abstraction, final manuscript approval; Tre D. Thorne: data abstraction; final manuscript approval; Jamie Conklin: search strategy design, search and database management, manuscript drafting, final manuscript approval; Andrea K. Knittel: review conceptualization, data abstraction, manuscript drafting, final manuscript approval, supervision.

## FUNDING INFORMATION

This project was supported in part by funds from the National Institute of Child Health and Human Development (NICHD) (Knittel, K12HD103085, PI Neal‐Perry) and the University of Minnesota Department of Obstetrics and Gynecology (Wise).

## CONFLICT OF INTEREST STATEMENT

The authors report no conflicts of interest.

## Supporting information


**File S1:** Full Search Strategy.

## Data Availability

Data sharing is not applicable to this article as no new data were created or analyzed in this study.

## References

[1] Fair H , Walmsley R . World Female Imprisonment List: Institute for Crime & Justice Policy Research; 2022. https://www.icpr.org.uk/news‐events/2022/world‐female‐prison‐population‐60‐2000

[2] Kajstura A , Sawyer W . Women's Mass Incarceration: The Whole Pie 2023: Prison Policy Initiative; 2023. https://www.prisonpolicy.org/reports/pie2023women.html

[3] National Institute of Justice . Correctional Facilities: United States Government, Department of Justice. https://nij.ojp.gov/topics/corrections/correctional‐facilities#:~:text=Institutional%20corrections%20facilities%20include%20prisons,typically%20longer%20than%20a%20year

[4] American College of Obstetricians and Gynecologists ACOG practice bulletin No. 141: management of menopausal symptoms. Obstet Gynecol. 2014;123(1):202‐216. 24463691 10.1097/01.AOG.0000441353.20693.78

[5] American College of Obstetricians and Gynecologists Committee on health Care for Underserved Women . ACOG Committee Opinion No. 830: reproductive health care for incarcerated pregnant, postpartum, and nonpregnant individuals. Obstet Gynecol. 2021;138(1):e24‐e34. 33906198 10.1097/AOG.0000000000004429

[6] Allsworth JE , Clarke J , Peipert JF , Hebert MR , Cooper A , Boardman LA . The influence of stress on the menstrual cycle among newly incarcerated women. Womens Health Issues. 2007;17(4):202‐209. 17560123 10.1016/j.whi.2007.02.002 PMC2170522

[7] Brousseau EC , Ahn S , Matteson KA . Cervical cancer screening access, outcomes, and prevalence of dysplasia in correctional facilities: a systematic review. J Womens Health (Larchmt). 2019;28(12):1661‐1669. 30939063 10.1089/jwh.2018.7440 PMC6919241

[8] McDaniels‐Wilson C , Belknap J . The extensive sexual violation and sexual abuse histories of incarcerated women. Violence Against Women. 2008;14(10):1090‐1127. 18757348 10.1177/1077801208323160

[9] Brousseau EC , Montplaisir R , Zeyl V , Has P . Comparing perceptions of long‐acting reversible contraception among women during periods of incarceration and women attending a local clinic: an exploratory study. Contraception. 2022;110:61‐65. 34971607 10.1016/j.contraception.2021.12.007

[10] Kirubarajan A , Tsang J , Dong S , et al. Pregnancy and childbirth during incarceration: a qualitative systematic review of lived experiences. BJOG. 2022;129(9):1460‐1472. 35274810 10.1111/1471-0528.17137

[11] Alirezaei S , Latifnejad Roudsari R . The needs of incarcerated pregnant women: a systematic review of literature. Int J Community Based Nurs Midwifery. 2022;10(1):2‐17. 35005037 10.30476/IJCBNM.2021.89508.1613 PMC8724729

[12] Hessami K , Hutchinson‐Colas JA , Chervenak FA , et al. Prenatal care and pregnancy outcome among incarcerated pregnant individuals in the United States: a systematic review and meta‐analysis. J Perinat Med. 2023;51(5):600‐606. 36394545 10.1515/jpm-2022-0412

[13] Spaulding AC , Rabeeah Z , Del Mar Gonzalez‐Montalvo M , et al. Prevalence and Management of Sexually Transmitted Infections in correctional settings: a systematic review. Clin Infect Dis. 2022;74(Suppl_2):S193‐S217. 35416974 10.1093/cid/ciac122 PMC9989347

[14] Peart MS , Knittel AK . Contraception need and available services among incarcerated women in the United States: a systematic review. Contracept Reprod Med. 2020;5:2. 32194976 10.1186/s40834-020-00105-w PMC7077150

[15] Paynter M , Pinzon Hernandez P , Heggie C , McKibbon S , Munro S . Abortion and contraception for incarcerated people: a scoping review. PLoS One. 2023;18(3):e0281481. 36996087 10.1371/journal.pone.0281481 PMC10062621

[16] Escobar N , Plugge E . Prevalence of human papillomavirus infection, cervical intraepithelial neoplasia and cervical cancer in imprisoned women worldwide: a systematic review and meta‐analysis. J Epidemiol Community Health. 2020;74(1):95‐102. 31649041 10.1136/jech-2019-212557

[17] Tricco AC , Lillie E , Zarin W , et al. PRISMA Extension for Scoping Reviews (PRISMA‐ScR): checklist and explanation. Ann Intern Med. 2018;169(7):467‐473. 30178033 10.7326/M18-0850

[18] Aday LA , Andersen R . A framework for the study of access to medical care. Health Serv Res. 1974;9(3):208‐220. 4436074 PMC1071804

[19] Saadi A , De Trinidad Young ME , Patler C , Estrada JL , Venters H . Understanding US immigration detention: reaffirming rights and addressing social‐structural determinants of health. Health Hum Rights. 2020;22(1):187‐197. 32669800 PMC7348446

[20] Acheampong LK , Effah K , Amuah JE , et al. Determining the prevalence of high‐risk human papillomavirus infection using a novel cervical precancer screening approach in incarcerated women at the Nsawam Medium Security Prison, Ghana. Ecancermedicalscience. 2021;15:1248. 34267804 10.3332/ecancer.2021.1248 PMC8241459

[21] Adamopoulos DA , Ierapetritakis G , Kapolla N . Pituitary function in young women in a reformatory institution. Br J Obstet Gynaecol. 1982;89(7):511‐515. 6807338 10.1111/j.1471-0528.1982.tb03650.x

[22] Aday R , Farney L . Malign neglect: assessing older women's health care experiences in prison. J Bioeth Inq. 2014;11(3):359‐372. 24990453 10.1007/s11673-014-9561-0

[23] Alcivar JC , Zambrano MM , Madroñero MG , et al. Sexually transmitted infections in inmates in merida Venezuela. Invest Clin (Venezuela). 2020;61(3):227‐241.

[24] Alonso‐Sanz M , Chaves F , Sánchez S , Romero N , Dronda F . [Microbiological study of some microorganisms implicated in sexually transmitted diseases among the female prison population]. Enferm Infecc Microbiol Clin. 1996;14(8):474‐478. 9011204

[25] Alward HC . Gynecologic problems in an institution for the management of juvenile delinquency. Some general experiences at Juvenile Hall. Am J Obstet Gynecol. 1966;95(3):366‐373. 5939258 10.1016/0002-9378(66)90118-9

[26] Andrade FM , Andrade SG , Souza R , Samaja T . EP28.13: gynecological ultrasound of patients in prison. Ultrasound Obstet Gynecol. 2017;50:391.

[27] Artymuk NV , Marochko KV . Efficiency of human papillomavirus detection with a vaginal discharge self‐collection device. Akusherstvo i Ginekologiya (Russian Federation). 2016;3:85‐91.

[28] Artymuk N , Marochko K . The prevalence of human papillomavirus infection among female prisoners in Siberia. Eur J Contracept Reprod Health Care. 2016;21:133.

[29] Arua MC , Nnam MU , Arua CC , et al. ‘…Because We're the minority, We're neglected’: exploring gender‐specific problems facing female inmates in a male custodial Centre. J Aggress Maltreat Trauma. 2023;33: 179‐199.

[30] Binswanger IA , Krueger PM , Steiner JF . Prevalence of chronic medical conditions among jail and prison inmates in the USA compared with the general population. J Epidemiol Community Health. 2009;63(11):912‐919. 19648129 10.1136/jech.2009.090662

[31] Bozelko C , Bobel C , Winkler IT , Fahs B , Hasson KA , Kissling EA . Opinion: prisons that withhold menstrual pads humiliate women and violate basic rights. The Palgrave Handbook of Critical Menstruation Studies. Springer Singapore; 2020:49‐51. 33347166

[32] Butler T , Donovan B , Taylor J , et al. Herpes simplex virus type 2 in prisoners, New South Wales, Australia. Int J STD AIDS. 2000;11(11):743‐747. 11089789 10.1258/0956462001915174

[33] Caloenescu M , Larose G , Birry A , Roy J , Kasatiya SS . Genital infection in juvenile delinquent females. Br J Vener Dis. 1973;49(1):72‐77. 4632812 10.1136/sti.49.1.72 PMC1044857

[34] Cañadas MP , Martínez F , de Sanjosé S , et al. [Detection of human papillomavirus DNA by PCR in high‐risk women. Validation of a protocol]. Enferm Infecc Microbiol Clin. 1998;16(9):400‐403. 9887625

[35] Carlen P . Women's Imprisonment: A Study in Social Control. Routledge; 2021.

[36] Carter M . Knowledge about sexual matters in delinquent girls. Br J Psychiatry. 1969;115(519):221‐224. 5778199 10.1192/bjp.115.519.221

[37] Celia Bórquez B , Teresa Reyes R , Hilda Villanueva D , Carlos Soto S , Mariana León G , Claudio Andres Alburquenque O . Prevalence of sexually transmitted infections and vaginal infections in women inmates of a prison in Arica city. Rev Chilena Infectol. 2022;39(4):421‐431.

[38] Cerqueira EM , Santoro CL , Donozo NF , et al. Genetic damage in exfoliated cells of the uterine cervix. Association and interaction between cigarette smoking and progression to malignant transformation? Acta Cytol. 1998;42(3):639‐649. 9622681 10.1159/000331820

[39] Chirwa VM . Prisons in Malawi. 2001.

[40] Chirwa VM . Report of the Mission of the Special Rapporteur on Prisons and Conditions of Detention in Africa to the Federal Democratic Republic of Ethiopia. 2004.

[41] Chirwa VM . Uganda: Mission on Prisons and Conditions of Detention. 2001.

[42] Clarke JG , Hebert MR , Rosengard C , Rose JS , DaSilva KM , Stein MD . Reproductive health care and family planning needs among incarcerated women. Am J Public Health. 2006;96(5):834‐839. 16571701 10.2105/AJPH.2004.060236 PMC1470599

[43] Coker AL , Patel NJ , Krishnaswami S , Schmidt W , Richter DL . Childhood forced sex and cervical dysplasia among women prison inmates. Violence Against Women. 1998;4(5):595‐608.

[44] D'Alessandro B , Gasbarro R , Montano A , Bellastella A . [On the adreno‐ovarian androgenic function in women in prison. I. (preliminary note)]. Quad Criminol Clin. 1972;14(3):329‐338. 4276764

[45] D'Eça JA , Cunha SF , Costa MR , de Sousa VEC , Soares DL , Mochel EG . Uterine cervical cancer: a study comprised of women in prison. J Nurs UFPE/Revista de Enfermagem UFPE. 2011;5(9):2175‐2181.

[46] de Araújo PF , Kerr L , Kendall C , et al. Behind bars: the burden of being a woman in Brazilian prisons. BMC Int Health Hum Rights. 2020;20(1):28. 33121484 10.1186/s12914-020-00247-7 PMC7594946

[47] de Cássia Ferreira R , Vidal Pereira A , Herdy Alves V , dos Vieira Santo M , Pereira Rodrigues D , Soanno Marchiori GR . Health status of climacteric women in the prison system. Cogitare Enferm. 2017;22(1):1‐7.

[48] de Insuasty MB , Erazo JV , Alvarez AM , Casas MI , de Collazos OO , Álvarez‐Soler J . Prevalence of cervical cytological abnormalities on three population groups of women in Popayán, Colombia 2003–2005. Rev Colomb Obstet Ginecol. 2008;59(3):190‐198.

[49] De Jesús Cabrera López T , Ramos‐Alamillo U , Palacios CC , González‐Rodríguez A , Naves EL , Díaz S . HIV/STI prevalence and sociodemographic data in incarcerated female adolescents in Mexico City. J Int AIDS Soc. 2015;18:14.

[50] De Sanjosé S , Bosch FX , Valls I , et al. Prevalence of HPV cervical infections among imprisoned women in Barcelona, Spain. Sex Transm Infect. 2000;76(1):58. 10.1136/sti.76.1.58 PMC1760560 10817075

[51] de Sousa‐Rodrigues CF , Gusmão LCB , Pereira GO , Bárbara GHS , Júnior DLB , de Jesus SRR . Prevalência e gravidade de sintomas da síndrome pré‐menstrual em reeducandas condenadas por crimes violentos [Prevalence and severity of premenstrual syndrome symptoms in arrested women condemned by violent crimes]. J Bras Psiquiatr. 2006;55(1):58‐61.

[52] Deboscker F , Nacher M , Adenis A , et al. Sexual and reproductive health of incarcerated women in French Guiana: a qualitative approach. Int J Prison Health. 2022;18(4):371‐383. 34784119 10.1108/IJPH-05-2021-0039

[53] Delage de Luget C , Jauffret C , Faust C , Knight S , Bartoli C , Ricard E . Cervical dysplasia and treatments barrier in jail: a study in Marseille's Detention Center‐Les Baumettes, France. Womens Health Rep (New Rochelle). 2022;3(1):670‐677. 36147834 10.1089/whr.2021.0135 PMC9436383

[54] Di Giuseppe G , Folcarelli L , Lanzano R , Napolitano F , Pavia M . HPV vaccination and cervical cancer screening: assessing awareness, attitudes, and adherence in detained women. Vaccines (Basel). 2022;10(8):1280. 36016168 10.3390/vaccines10081280 PMC9416201

[55] dos Anjos SJ , Ribeiro SG , Lessa PR , Nicolau AI , Vasconcelos CT , Pinheiro AK . [Risk factors for cancer of the cervix in women prisoners]. Rev Bras Enferm. 2013;66(4):508‐513. 24008703 10.1590/s0034-71672013000400007

[56] Drennan V , Goodman C , Norton C , Wells A . Incontinence in women prisoners: an exploration of the issues. J Adv Nurs. 2010;66(9):1953‐1967. 20626478 10.1111/j.1365-2648.2010.05377.x

[57] Drennan V , Goodman C , Norton C , Wells A . Incontinence: enhancing care in women's prisons. Nurs Times. 2011;107(17):18‐19. 21614954

[58] Emerson A , Allison M , Kelly PJ , Ramaswamy M . Barriers and facilitators of implementing a collaborative HPV vaccine program in an incarcerated population: a case study. Vaccine. 2020;38(11):2566‐2571. 32046888 10.1016/j.vaccine.2020.01.086 PMC7133786

[59] Emerson A , Allison M , Saldana L , Kelly PJ , Ramaswamy M . Collaborating to offer HPV vaccinations in jails: results from a pre‐implementation study in four states. BMC Health Serv Res. 2021;21(1):309. 33827560 10.1186/s12913-021-06315-5 PMC8028758

[60] Emerson AM , Smith S , Lee J , Kelly PJ , Ramaswamy M . Effectiveness of a Kansas City, jail‐based intervention to improve cervical health literacy and screening, one‐year post‐intervention. Am J Health Promot. 2020;34(1):87‐90. 31315420 10.1177/0890117119863714 PMC6920557

[61] Farhoudi B , Shahmohamadi E , SeyedAlinaghi S , et al. Prevalence of sexually transmitted infections (STIs) and related factors among female prisoners in Tehran, Iran. Int J Prison Health. 2023;19(4):492‐500. 36576269 10.1108/IJPH-09-2022-0055

[62] Farley JL , Mitty JA , Lally MA , et al. Comprehensive medical care among HIV‐positive incarcerated women: the Rhode Island experience. J Womens Health Gend Based Med. 2000;9(1):51‐56. 10718506 10.1089/152460900318966

[63] Fink MJ , Goodman A , Hight E , Miller‐Mack E , de Groot A . Critical prevention, critical care: gynecological and obstetrical aspects of comprehensive HIV prevention and treatment among incarcerated women. J Correct Health Care. 1998;5(2):201‐223.

[64] Fontebo HN . Prison Conditions in Cameroon: The Narratives of Female Inmates. 2013.

[65] Friedman E , Burr E , Sufrin C . Seeking recognition through carceral health care bureaucracy: analysis of medical care request forms in a county jail. Soc Sci Med. 2021;291. 10.1016/j.socscimed.2021.114485 PMC8756140 34662761

[66] Gallagher E . Genital infection in young delinquent girls. Br J Vener Dis. 1970;46(2):129‐131. 5468129 10.1136/sti.46.2.129 PMC1048045

[67] Gallagher CA , Dobrin A , Douds AS . A national overview of reproductive health care services for girls in juvenile justice residential facilities. Womens Health Issues. 2007;17(4):217‐226. 17602966 10.1016/j.whi.2007.02.006

[68] Garcia A , Exposto F , Prieto E , Lopes M , Duarte A , Correia da Silva R . Association of Trichomonas vaginalis with sociodemographic factors and other STDs among female inmates in Lisbon. Int J STD AIDS. 2004;15(9):615‐618. 15339370 10.1258/0956462041724235

[69] Gender Health & Justice Research Unit . Women in Prison: Health and Mental Health. 2012.

[70] Getachew M , Haile D , Churko C , Gube AA . Magnitude of self‐reported syndromes of sexually transmitted infections and its associated factors among young incarcerated persons (18–29 years) in correctional facilities of Gamo Gofa zone, Southern Ethiopia. Risk Manag Healthc Policy. 2021;14:21‐29. 33442313 10.2147/RMHP.S285289 PMC7797331

[71] Goodman M , Dawson R , Burlingame P . Reproductive Health behind Bars in California. American Civil Liberties Union of California; 2016;24:2017. Accessed January. www.acluncorg/ReproductiveHealthBehindBars_Report.

[72] Grady C . Hot flashes in the cooler: menopausal women in a U.S. prison. Friend Indeed: For Women in the Prime of Life. 1999;16(5):1‐4.

[73] Gullberg S . State of the Estate‐Women in Prison's Report on the women's Custodial Estate 2011–12. Women in Prison; 2013.

[74] Haller DM , Sebo P , Cerutti B , et al. Primary care services provided to adolescents in detention: a cross‐sectional study using ICPC‐2. Acta Paediatr. 2010;99(7):1060‐1064. 20178509 10.1111/j.1651-2227.2010.01716.x

[75] Haller DM , Sebo P , Bertrand D , Cerutti B , Eytan A , Wolff H . Primary care services provided to adolescents in detention: a study in a juvenile detention facility in Switzerland. J Adolesc Health. 2009;44(2):S31‐S32.

[76] Harner H , Burgess AW . Using a trauma‐informed framework to care for incarcerated women. J Obstet Gynecol Neonatal Nurs. 2011;40(4):469‐475; quiz 75. 10.1111/j.1552-6909.2011.01259.x 21894637

[77] Hatton DC , Kleffel D , Fisher AA . Prisoners' perspectives of health problems and healthcare in a US women's jail. Women Health. 2006;44(1):119‐136. 17182530 10.1300/J013v44n01_07

[78] Henderson CE , Rich JD , Lally MA . HPV vaccination practices among juvenile justice facilities in the United States. J Adolesc Health. 2010;46(5):495‐498. 20413087 10.1016/j.jadohealth.2009.10.007 PMC2927822

[79] Holland‐Hall CM , Wiesenfeld HC , Murray PJ . Self‐collected vaginal swabs for the detection of multiple sexually transmitted infections in adolescent girls. J Pediatr Adolesc Gynecol. 2002;15(5):307‐313. 12547662 10.1016/s1083-3188(02)00197-3

[80] Hyde R , Brumfield B , Nagel J . Female inmate health care requests. J Correct Health Care. 2000;7(1):91‐103.

[81] Ingram‐Fogel C . Health problems and needs of incarcerated women. J Prison Jail Health. 1991;10(1):43‐57.

[82] Ishihara K . A study of menstrual distress. Jpn J Crim Psychol. 1986;24(1):13‐25.

[83] Jaffe EF , Palmquist AEL , Knittel AK . Experiences of menopause during incarceration. Menopause. 2021;28(7):829‐832. 33739317 10.1097/GME.0000000000001762 PMC8495613

[84] Jaffe E , Rosen D , Palmquist A , Knittel AK . Menopause‐related medication use among women age 45‐75 experiencing incarceration in North Carolina 2015‐2016. Int J Prison Health. 2022;18(2):176‐184. 38899606 10.1108/IJPH-07-2021-0068

[85] Jiamset I , Sakdejayont S , Rattanakhot N , Peeyananjarassri K , Dechaphunkul A , Sunpaweravong P . Cervical cancer screening in incarcerated women: an experience from the first cervical cancer screening campaign in a southern Thailand correctional facility. J Clin Oncol. 2019;37:6572.

[86] Kanbergs AN , Sullivan MW , Maner M , et al. Cervical cancer screening and follow‐up practices in U.S. prisons. Am J Prev Med. 2023;64(2):244‐249. 36653100 10.1016/j.amepre.2022.09.021

[87] Keighley EE . Trichomoniasis in a closed community: efficacy of metronidazole. Br Med J. 1971;1(5742):207‐209. 5099972 10.1136/bmj.1.5742.207 PMC1794842

[88] Kelly PJ , Driscoll D , Lipnicky A , Anderson S , Glenn J , Ramaswamy M . Developing a cancer prevention health education resource: a primer of process and evaluation. J Cancer Educ. 2022;37(2):274‐279. 32583352 10.1007/s13187-020-01807-0 PMC7759588

[89] Kelly PJ , Hunter J , Daily EB , Ramaswamy M . Challenges to pap smear follow‐up among women in the criminal justice system. J Community Health. 2017;42(1):15‐20. 27449030 10.1007/s10900-016-0225-3 PMC5253085

[90] Kesha K , O'Donnell L , Kapoor P , Chacho M . Papanicolaou test results in a cytology laboratory: what does it take to cause a shift? Am J Clin Pathol. 2012;138:A269.

[91] Kim S , Richardson S , Puisis M , Chakrabarti S , Davis F . Cervical cancer screening in an urban jail. Am J Epidemiol. 2011;173:S156.

[92] Kim S , Richardson T , Rodriguez S , Davis F . Cervical Cancer Screening in a Large Urban Jail: Six‐Year Report. 2000–2005. 134th Annual Meeting and Exposition of the American Public Health Association 2006.

[93] Klobusický M , Gombosová A , Demes P , Fabusová H , Jánoska A . [Urogenital trichomoniasis and vaginal mycosis in female offenders—a repeat study 20 years later]. Bratisl Lek Listy. 1989;90(9):700‐704. 2590856

[94] Kouyoumdjian FG , McConnon A , Herrington ERS , Fung K , Lofters A , Hwang SW . Cervical cancer screening access for women who experience imprisonment in Ontario, Canada. JAMA Netw Open. 2018;1(8):e185637. 30646279 10.1001/jamanetworkopen.2018.5637 PMC6324332

[95] Kraft‐Stolar T . Reproductive Injustice: The State of Reproductive Health Care for Women in New York State Prisons. 2015.

[96] Lee CD . Social significance of genital tract infections in adolescent girls admitted to a juvenile remand centre. Med J Aust. 1987;146(5):277‐278. 3821624 10.5694/j.1326-5377.1987.tb120243.x

[97] Leite Araújo MA , de Assis Araújo Fernans E , Lima Barros V , Freitas Amorim R . Behavioral and Infractional aspects of female teenagers deprived of liberty. Texto Contexto Enferm. 2019;28:1‐14.

[98] Lipnicky A , Stites S , Sufrin C , et al. Jail provision of pregnancy and sexual health services in four midwestern states. Womens Health Issues. 2023;33(1):97‐104. 36096980 10.1016/j.whi.2022.07.004

[99] Litt IF , Cohen MI . Age of menarche: a changing pattern and its relationship to ethnic origin and delinquency. J Pediatr. 1973;82(2):288‐289. 4684373 10.1016/s0022-3476(73)80171-4

[100] López‐Barbosa N , Castro‐Jiménez MA , Gamboadelgado EM , Vera‐Cala LM . Prevalence and determinants of vaginal infections in women imprisoned in a Colombian jail. Rev Chil Obstet Ginecol. 2009;74(2):77‐82.

[101] Lowery CL , Bronstein JM , Benton TL , Fletcher DA . Distributing medical expertise: the evolution and impact of telemedicine in Arkansas. Health Aff (Millwood). 2014;33(2):235‐243. 24493766 10.1377/hlthaff.2013.1001

[102] Luyt WFM , Du Preez N . A case study of female incarceration in South Africa. Acta Criminol: Afr J Criminol Victimol. 2010;23(3):88‐114.

[103] Mahmood S , Imran A , Hussain S , Iqbal N , Shabeer Gorya I , Arsalan Omer M . Frequency of urological problems and their management in prisoners of Kot‐Lakhpat Jail Lahore. Pak J Med Health Sci. 2020;14(2):538‐541.

[104] Mahto M , Zia S . Measuring the gap: from home office to the National Health Service in the provision of a one‐stop shop sexual health service in a female prison in the UK. Int J STD AIDS. 2008;19(9):586‐589. 18725547 10.1258/ijsa.2008.008051

[105] Mehta S , Singla A , Jadaun P . Cervical cancer screening behind bars: a woman's right. J Clin Diagn Res. 2020;14(11):9‐11.

[106] Michelsen J . Epidemiologic studies and early detection of cancer in the National Penitentiary for women in Bogota, D.E., Colombia. Rev Colomb Obstet Ginecol. 1969;20(5):341‐347. 5361320

[107] Miranda AE , Merçon‐de‐Vargas PR , Viana MC . Sexual and reproductive health of female inmates in Brazil. Rev Saude Publica. 2004;38(2):255‐260. 15122382 10.1590/s0034-89102004000200015

[108] Miranda AE , Vargas PM , St Louis ME , Viana MC . Sexually transmitted diseases among female prisoners in Brazil: prevalence and risk factors. Sex Transm Dis. 2000;27(9):491‐495. 11034522 10.1097/00007435-200010000-00001

[109] Modie‐Moroka T . Vulnerability across a life course: an empirical study: women and criminality in Botswana prisons. J Soc Dev Afr. 2003;18(1):145‐179.

[110] Moore A , Cox‐Martin M , Berenbaum K , Binswanger IA . Hpv vaccination in correctional care: knowledge, attitudes, barriers, and rates of vaccination among female inmates. J Gen Intern Med. 2017;32(2):S210.

[111] Moore A , Cox‐Martin M , Dempsey AF , Berenbaum Szanton K , Binswanger IA . HPV vaccination in correctional care: knowledge, attitudes, and barriers among incarcerated women. J Correct Health Care. 2019;25(3):219‐230. 31242811 10.1177/1078345819853286

[112] Morton JH , Additon H , Addison RG , Hunt L , Sullivan JJ . A clinical study of premenstrual tension. Am J Obstet Gynecol. 1953;65(6):1182‐1191. 13057949 10.1016/0002-9378(53)90358-5

[113] Muscat K , Cremona C , Melillo Fenech T , Abela M , Padovese V . Sexually transmitted infections epidemiology and risk assessment at the main correctional facility in Malta (2017‐2019). J Eur Acad Dermatol Venereol. 2022;36(1):113‐118. 34549833 10.1111/jdv.17681

[114] Nangia EN , Fontebo HN . Treatment of female offenders in prison: the case of Cameroon. Int J Humanit Soc Stud. 2017;5:318‐324.

[115] United Nations . Assessment of the Prison System in Mogadishu South 2012. 2012.

[116] Nicholson J , Almond L , Rizvi N , Fairley CK . Low prevalence of STIs among women in prison, but bacterial vaginosis is common. Aust N Z J Public Health. 2003;27(4):464‐466. 10.1111/j.1467-842x.2003.tb00428.x 14705313

[117] Nicolau AIO , de Souza Aquino P , Barbosa Ximenes L , Bezerra Pinheiro K . Proximal social determinants related to cervical cancer in imprisoned women. Rev Min Enferm. 2015;19(3):733‐742.

[118] Nicolau AIO , Ribeiro SS , Lessa PRA , Monte AS , Ferreira RCN , Pinheiro AKB . A picture of the socioeconomic and sexual reality of women prisoners. Acta Paul Enferm. 2012;25(3):386‐392.

[119] Nijhawan AE , Chapin KC , Salloway R , et al. Prevalence and predictors of trichomonas infection in newly incarcerated women. Sex Transm Dis. 2012;39(12):973‐978. 23191953 10.1097/OLQ.0b013e31826e8847 PMC3878291

[120] Office of the Inspectorate Te Tari Tirohia . Thematic Report: The Lived Experience of Women in Prison. 2021.

[121] Oliván Gonzalvo G . Health and nutritional status of delinquent female adolescents. An Esp Pediatr. 2002;56(2):116‐120. 11827672

[122] Pereira Borges A , Arenhardt K , Pereira Terças AC , et al. Socioeconomic and sexual profile of incarcerated women. J Nursing UFPE/Revista de Enfermagem UFPE. 2018;12(7):1978‐1985.

[123] Pereyra AJ . The relationship of sexual activity to cervical cancer. Cancer of the cervix in a prison population. Obstet Gynecol. 1961;17:154‐159. 13734313

[124] Philipp E . Gynaecology in prison. Br J Clin Pract. 1987;41(1):549‐552. 3663455

[125] Pickett ML , Allison M , Twist K , Klemp JR , Ramaswamy M . Breast cancer risk among women in jail. Biores Open Access. 2018;7(1):139‐144. 30250761 10.1089/biores.2018.0018 PMC6151327

[126] Plugge E , Douglas N , Fitzpatrick R . The Health of Women in Prison Study Findings. Department of Public Health University of Oxford; 2006.

[127] Centro pela Justiça e pelo Direito Internacional . Relatório sobre mulheres encarceradas no Brasil. 2007.

[128] Ribeiro de Menezes P , Teixeira Moreira Vasconcelos C , Gomes Lopes L , Macena de Almeida ME , Moura Barbosa Castro RC , Vasconcelos Neto JA . Lower urinary tract symptoms in female prison inmates: prevalence and impact on quality of life. Int Urogynecol J. 2021;32(10):2795‐2802. 33609160 10.1007/s00192-021-04679-0

[129] Ribeiro SG , Lessa PRA , Monte AS , et al. Gynecologic and obstetric profile of state imprisoned females. Texto Contexto. 2013;22(1):13‐21.

[130] Rice A , Thompson JA , Briffa K . Bladder and bowel symptoms following imprisonment in West Australian female prisons. Int J Prison Health. 2022;18(1):15‐26. 34259421 10.1108/IJPH-07-2020-0050

[131] Ris HW , Dodge RW . Trichomonas and yeast vaginitis in institutionalized adolescent girls. Wis Med J. 1973;72(6):150. 4713536

[132] Risser WL , Cromwell PF , Bortot AT , Risser JM . Impact of new diagnostic criteria on the prevalence and incidence of pelvic inflammatory disease. J Pediatr Adolesc Gynecol. 2004;17(1):39‐44. 15010038 10.1016/j.jpag.2003.11.012

[133] Robertson DH , George G . Medical and legal problems in the treatment of delinquent girls in Scotland. II. Sexually transmitted disease in girls in custodial institutions. Br J Vener Dis. 1970;46(1):46‐53. 5467198 10.1136/sti.46.1.46 PMC1048023

[134] Rowe AW , Waters MV . Physical associations in adults with behavior problems. Endocrinology. 1935;19(2):129‐143.

[135] Ruiz‐Maza JC , Soto‐Azpilcueta RA , Sanchez‐Salvatierra J , Torres‐Prado Y . Cytological screening for cervical cancer and associated factors in the penitentiary population of Peru. Rev Esp Sanid Penit. 2018;20(3):103‐110. 30908565 PMC6463325

[136] Sabbagh Steinberg NG . ‘It's Here, But You Can't Always Get To It’: The Experience of Women in Prison With Gynecological Care. 2019;80.

[137] Samakaya‐Makaratie J . Female Prisoners in ‘Male’ Prisons. A Tragedy of Lives: Women in Prison in Zimbabwe. 2003.

[138] Sarpong AA , Otupiri E , Yeboah‐Awudzi K , Osei‐Yeboah J , Berchie GO , Ephraim RKD . An assessment of female prisoners' perception of the accessibility of quality healthcare: a survey in the Kumasi central prisons, Ghana. Ann Med Health Sci Res. 2015;5(3):179‐184. 26097759 10.4103/2141-9248.157495 PMC4455007

[139] Schneider‐Reinkens A . On the treatment of climacteric in closed institutions. Z Allgemeinmed. 1969;45(18):870‐873. 5800261

[140] Singla A , Mehta S . Health behind bars: a woman's right. Int J Gynecol Cancer. 2022;32:A376‐A377.

[141] Smith RA , Andrews KS , Brooks D , et al. Cancer screening in the United States, 2019: a review of current American Cancer Society guidelines and current issues in cancer screening. CA Cancer J Clin. 2019;69(3):184‐210. 30875085 10.3322/caac.21557

[142] Stephens T , Holliday RC , Jarboe J . Self‐reported ecstasy (MDMA) use and past occurrence of sexually transmitted infections (STIs) in a cohort juvenile detainees in the USA. J Community Health. 2015;40(2):308‐313. 25160467 10.1007/s10900-014-9936-5

[143] Stevens J , Zierler S . Prevalence of prior sexual abuse and HIV risk‐taking behaviors in incarcerated women in Massachusetts. J Correct Health Care. 1995;2(2):137‐149.

[144] Sutcliffe S , Newman SB , Hardick A , Gaydos CA . Prevalence and correlates of trichomonas vaginalis infection among female US federal prison inmates. Sex Transm Dis. 2010;37(9):585‐590. 20803782 10.1097/olq.0b013e3181de4113 PMC4800006

[145] Tang A , Maw R , Kell P . A survey of sexual health services in UK prisons. Int J STD AIDS. 2010;21(9):638‐641. 21097737 10.1258/ijsa.2010.010177

[146] de Tavares Oliveira JL , Lessa Pacheco ZM , Arreguy Senna C . Vulnerabilidade de mulheres às infecções sexualmente transmissíveis e câncer de colo uterino em uma unidade prisional. Rev Aten Prim Salud. 2020;23(4):853‐872.

[147] Teck J , Beyda RM , Eissa M , Benjamins L . Immunizations within the context of juvenile detention. J Adolesc Health. 2016;58(2):S55.

[148] Todrys KW , Amon JJ . Health and human rights of women imprisoned in Zambia. BMC Int Health Hum Rights. 2011;11(1):1‐7. 21696625 10.1186/1472-698X-11-8 PMC3141661

[149] Topp SM , Moonga CN , Luo N , et al. Mapping the Zambian prison health system: an analysis of key structural determinants. Glob Public Health. 2017;12(7):858‐875. 27388512 10.1080/17441692.2016.1202298

[150] Topp SM , Moonga CN , Mudenda C , et al. Health and healthcare access among Zambia's female prisoners: a health systems analysis. Int J Equity Health. 2016;15(1):1‐14. 27671534 10.1186/s12939-016-0449-y PMC5037633

[151] Twea S . Women as Offenders—The Social and Legal Circumstances of Women Who Commit Crimes: A Case Study of Selected Prisons in Malawi. 2004.

[152] Valent M , Catár G , Klobusický M , Valentová M , Holková R , Solár G . Gynecological, venereal and parasitologic findings in delinquent girls. Bratisl Lek Listy. 1973;59(6):703‐708. 4741000

[153] Woodall J , Freeman C , Warwick‐Booth L . Health‐promoting prisons in the female estate: an analysis of prison inspection data. BMC Public Health. 2021;21(1):1‐8. 34418998 10.1186/s12889-021-11621-y PMC8380381

[154] Young DS . Health status and service use among incarcerated women. Fam Community Health. 1998;21:16‐31.

[155] Young VD , Reviere R . Meeting the health care needs of the new woman inmate. J Offender Rehabil. 2001;34(2):31‐48.

[156] Smith C . A period in custody: menstruation and the imprisoned body. Internet J Criminol. 2009;2009:1‐22.

[157] United Nations General Assembly . Basic principles for the treatment of prisoners (resolution 45/111). Office of the High Commissioner for Human Rights; 1990.

[158] Miller NA , Najavits LM . Creating trauma‐informed correctional care: a balance of goals and environment. Eur J Psychotraumatol. 2012;3(1). 10.3402/ejpt.v3i0.17246 PMC3402099 22893828

[159] Ahalt C , Haney C , Ekhaugen K , Williams B . Role of a US‐Norway exchange in placing health and well‐being at the center of US prison reform. Am J Public Health. 2020;110(S1):S27‐S29. 31967893 10.2105/AJPH.2019.305444 PMC6987915

[160] Hogben M , St. Lawrence JS . HIV/STD risk reduction interventions in prison settings. J Womens Health Gend Based Med. 2000;9(6):587‐592. 10957746 10.1089/15246090050118107

[161] Fogel CI , Crandell JL , Neevel AM , et al. Efficacy of an adapted HIV and sexually transmitted infection prevention intervention for incarcerated women: a randomized controlled trial. Am J Public Health. 2015;105(4):802‐809. 25211714 10.2105/AJPH.2014.302105 PMC4358199

[162] Pickett ML , Wickliffe J , Emerson A , Smith S , Ramaswamy M . Justice‐involved women's preferences for an internet‐based sexual health empowerment curriculum. Int J Prison Health. 2019;16(1):38‐44. 32040270 10.1108/IJPH-01-2019-0002 PMC7412955

[163] Williams BA , Stern MF , Mellow J , Safer M , Greifinger RB . Aging in correctional custody: setting a policy agenda for older prisoner health care. Am J Public Health. 2012;102(8):1475‐1481. 22698042 10.2105/AJPH.2012.300704 PMC3464842

