# Supplementary material for: Gynecologic conditions in the context of incarceration: A scoping review
Source: Int J Gynaecol Obstet. 2026 Feb 27;173(1):117–37. doi: 10.1002/ijgo.70873 (PMC12988401; doi:10.1002/ijgo.70873)
Supplement: Supplementary file 1 — File S1: Full Search Strategy. [file IJGO-173-117-s001.zip › PRISMA Diagram.docx]

**Identification of studies via other methods**

**Identification of studies via databases and registers**

Records identified from:

Websites (n = 0)

Organisations (n = 0)

Citation searching (n = 88)

etc.

Records removed *before screening*:

Duplicate records removed (n = 1182)

Records marked as ineligible by automation tools (n = 0)

Records removed for other reasons (n = 0)

Records identified from*:

Databases (n = 3329)

APA PsycInfo (n = 400)

CINAHL (n = 325)

Embase (n = 1100)

PubMed (n = 853)

Scopus (n = 651)

Registers (n = 3)

**Identification**

Records screened

(n = 2150)

Records excluded

(n = 1519)

Reports not retrieved

(n = 2)

Reports sought for retrieval

(n = 70)

Reports sought for retrieval

(n = 631)

Reports not retrieved

(n = 11)

**Screening**

Reports excluded:

Commentary only (n = 32)

Concentration camp (n = 22)

Erratum (n = 2)

Incarcerated organ (n = 5)

Included in a previous review (n = 48)

Medical humanities (n = 22)

Menstruation as an explanation of criminal behavior (n = 27)

Not about incarceration (n = 168)

Review (n = 25)

Sexual assault (n = 5)

STI/pregnancy (n = 64)

Trial registration (n = 2)

Wrong condition (n = 82)

Reports excluded:

Commentary only (n = 2)

Included in a review (n = 5)

Not about incarceration (n = 4)

Review (n = 10)

Sexual assault (n = 1)

STI/pregnancy (n = 9)

Wrong condition (n = 16)

Reports assessed for eligibility

(n = 68)

Reports assessed for eligibility

(n = 620)

Studies included in review

(n = 135)

Reports of included studies

(n = 137)

**Included**
