# Supplementary material for: Gynecologic conditions in the context of incarceration: A scoping review
Source: Int J Gynaecol Obstet. 2026 Feb 27;173(1):117–37. doi: 10.1002/ijgo.70873 (PMC12988401; doi:10.1002/ijgo.70873)
Supplement: Supplementary file 1 — File S1: Full Search Strategy. [file IJGO-173-117-s001.zip › Search Strategy_Supplementary.pdf]

Search Strategy Report:

Database: PubMed

Set #	
1	"Correctional Facilities"[Mesh] OR "Prisoners"[Mesh] OR "Criminals"[Mesh] OR "Juvenile Delinquency"[Mesh] OR “mass incarceration”[tw] OR "incarcerated women"[tw] OR "incarcerated people"[tw] OR "incarcerated persons"[tw] OR "women incarcerated"[tw] OR "incarcerated youths"[tw] OR "incarcerated adolescent"[tw] OR "incarcerated adolescents"[tw] OR “adolescents incarcerated”[tw] OR "incarcerated girl"[tw] OR "incarcerated girls"[tw] OR "incarcerated patient"[tw] OR "incarcerated patients"[tw] OR “incarcerated individuals”[tw] OR “incarcerated populations”[tw] OR “incarcerated population”[tw] OR “incarcerated adults”[tw] OR “incarcerated adults”[tw] OR “incarcerated female”[tw] OR “incarcerated females”[tw] OR carceral[tw] OR "correctional facility"[tw] OR "correctional facilities"[tw] OR "correctional institution"[tw] OR "correctional institutions"[tw] OR "correctional center"[tw] OR "correctional centers"[tw] OR "correctional centre"[tw] OR "correctional centres"[tw] OR "correction facility"[tw] OR "correction facilities"[tw] OR "correction institution"[tw] OR "correction institutions"[tw] OR "correction center"[tw] OR "correction centers"[tw] OR "correction centre"[tw] OR "correction centres"[tw] “correctional health”[tw] OR “correctional healthcare”[tw] OR “correctional care”[tw] OR prisoner[tw] OR prisoners[tw] OR prison[tw] OR prisons[tw] OR imprisoned[tw] OR jail[tw] OR jails[tw] OR jailed[tw] OR inmate[tw] OR inmates[tw] OR criminal[tw] OR criminals[tw] OR felon[tw] OR felons[tw] OR offender[tw] OR offenders[tw] OR convict[tw] OR convicts[tw] OR "penal institution"[tw] OR "penal institutions"[tw] OR "penal facility"[tw] OR "penal facilities"[tw] OR penitentiary[tw] OR penitentiaries[tw] OR “justice involved”[tw] OR justice-involved[tw] OR “detention centers”[tw] OR “detention center”[tw] OR “detention centres”[tw] OR “detention centre”[tw] OR “detention facility”[tw] OR “detention facilities”[tw] OR “juvenile detention”[tw] OR “youth detention”[tw] OR “juvenile delinquency”[tw] OR “juvenile justice”[tw]
2	"Gynecology"[Mesh] OR "Gynecological Examination"[Mesh] OR "Lower Urinary Tract Symptoms"[Mesh] OR "Papanicolaou Test"[Mesh] OR "Genital Diseases, Female"[Mesh] OR gynecology[tw] OR gynaecology[tw] OR gynecologic[tw] OR gynaecologic[tw] OR gynecological[tw] OR gynaecological[tw] OR gynecologist[tw] OR gynaecologist[tw] OR gynecologists[tw] OR gynaecologists[tw] OR urogynecologic[tw] OR urogynaecologic[tw] OR urogynecological[tw] OR urogynaecological[tw] OR urogynecology[tw] OR urogynaecology[tw] OR OBGYN[tw] OR OB-GYN[tw] OR “female genital disease”[tw] OR “female genital diseases”[tw] OR “adnexal disease”[tw] OR “adnexal diseases”[tw] OR “adnexa disease”[tw] OR “adnexa diseases”[tw] OR “vaginal disease”[tw] OR “vaginal diseases”[tw] OR “vulvar disease”[tw] OR “vulvar diseases”[tw] OR “vulvovaginal disease”[tw] OR “vulvovaginal diseases”[tw] OR “vulvar diseases”[tw] OR “ovarian

	disease”[tw] OR “ovarian diseases”[tw] OR “ovarian cysts”[tw] OR “ovarian cyst”[tw] OR “Polycystic Ovary Syndrome”[tw] OR “uterine disease”[tw] OR “uterine diseases”[tw] OR “endometrial disease”[tw] OR “endometrial diseases”[tw] OR “cervical disease”[tw] OR “cervical diseases”[tw] OR vaginitis[tw] OR vulvovaginitis[tw] OR vulvitis[tw] OR vaginosis[tw] OR yeast[tw] OR candida[tw] OR Candidiasis[tw] OR “vaginal discharge”[tw] OR “vaginal discharges”[tw] OR “vaginal examination”[tw] OR “vaginal examinations”[tw] OR “vaginal exam”[tw] OR “vaginal exams”[tw] OR “pelvic examination”[tw] OR “pelvic examinations”[tw] OR “pelvic exam”[tw] OR “pelvic exams”[tw] OR Papanicolaou[tw] OR “pap test”[tw] OR “pap tests”[tw] OR “pap smear”[tw] OR “pap smears”[tw] OR “pap stain”[tw] OR “pap stains”[tw] OR “cervical smear”[tw] OR “cervical smears”[tw] OR “lower urinary tract symptoms”[tw]
3	"Uterine Hemorrhage"[Mesh] OR "uterine hemorrhage"[tw] OR "uterine hemorrhages"[tw] OR "uterine hemorrhaging"[tw] OR "uterus hemorrhage"[tw] OR "uterus hemorrhages"[tw] OR "uterus hemorrhaging"[tw] OR "uterine haemorrhage"[tw] OR "uterine haemorrhages"[tw] OR "uterine haemorrhaging"[tw] OR "uterus haemorrhage"[tw] OR "uterus haemorrhages"[tw] OR "uterus haemorrhaging"[tw] OR “uterine bleeding”[tw] OR “uterus bleeding”[tw] OR "vaginal hemorrhage"[tw] OR "vaginal hemorrhages"[tw] OR "vaginal hemorrhaging"[tw] OR "vaginal haemorrhage"[tw] OR "vaginal haemorrhages"[tw] OR "vaginal haemorrhaging"[tw] OR “vaginal bleeding”[tw] OR "vagina hemorrhage"[tw] OR "vagina hemorrhages"[tw] OR "vagina hemorrhaging"[tw] OR "vagina haemorrhage"[tw] OR "vagina haemorrhages"[tw] OR "vagina haemorrhaging"[tw] OR “vagina bleeding”[tw] OR “abnormal bleeding”[tw] OR “irregular bleeding”[tw] OR “abnormal menstrual bleeding”[tw] OR “abnormal menses”[tw] OR “Intermenstrual Bleeding”[tw] OR “Dysfunctional Uterine Bleeding”[tw] OR “Breakthrough Bleeding”[tw] OR “Bleeding Between Periods”[tw] OR “bleeding between menses”[tw] OR “heavy menstrual bleeding”[tw] OR “heavy menstruation”[tw] OR “heavy period”[tw] OR “heavy periods”[tw] OR “heavy menses”[tw] OR hypermenorrhea[tw] OR hypermenorrhoea[tw] OR menorrhagia[tw] OR metrorrhagia[tw] OR menometrorrhagia[tw] OR “irregular menstrual bleeding”[tw] OR “irregular menses”[tw]
4	"Menopause"[Mesh] OR menopaus*[tw] OR premenopaus*[tw] OR postmenopaus*[tw] OR perimenopause*[tw] OR “hot flashes”[tw] OR “hot flushes”[tw]
5	"Pelvic Pain"[Mesh] OR dysmenorrhea[tw] OR dysmenorrheas[tw] OR “piriformis muscle syndrome”[tw] OR “piriformis syndrome”[tw] OR endometriosis[tw] OR endometrioses[tw] OR (("Pain"[Mesh] OR "Pain Management"[Mesh] OR pain*[tw]) AND ("Pelvis"[Mesh] OR pelvis[tw] OR pelvic[tw]))

6	"Genital Neoplasms, Female"[Mesh] OR “Squamous Intraepithelial Lesions of the Cervix”[tw] OR (("Genitalia, Female"[Mesh] OR “female genital”[tw] OR “female genitals”[tw] OR “female genitalia”[tw] OR “female reproductive”[tw] OR Gynecologic[tw] OR cervical[tw] OR cervix[tw] OR endometrium[tw] OR endometria[tw] OR endometrial[tw] OR ovary[tw] OR ovaries[tw] OR ovarian[tw] OR uterus[tw] OR uteri[tw] OR womb[tw] OR wombs[tw] OR uterine[tw] OR vagina[tw] OR vaginas[tw] OR vaginal[tw] OR vulva[tw] OR vulvas[tw] OR vulvar[tw] OR “fallopian tube”[tw] OR “fallopian tubes”[tw]) AND ("Neoplasms"[Mesh] OR neoplasm[tw] OR neoplasms[tw] OR neoplasia[tw] OR cancer[tw] OR cancers[tw] OR cancerous[tw] OR carcinoma[tw] OR carcinomas[tw] OR malignant[tw] OR malignancy[tw] OR malignancies[tw] OR metastasis[tw] OR metastases[tw] OR metastatic[tw] OR tumor[tw] OR tumors[tw] OR tumour[tw] OR tumours[tw] OR sarcoma[tw] OR sarcomas[tw] OR oncology[tw] OR oncologist[tw] OR oncologists[tw] OR oncologica[tw]))
7	("Pelvic Organ Prolapse"[Mesh] OR (("Prolapse"[Mesh] OR prolapse[tw] OR prolapses[tw]) AND ("Pelvis"[Mesh] OR “Vagina”[Mesh] OR "Uterus"[Mesh] OR pelvis[tw] OR pelvic[tw] OR uterine[tw] OR visceral[tw] OR rectal[tw] OR vaginal[tw] OR vaginal[tw] OR urogenital[tw] OR genital[tw] OR urinary[tw] OR genitourinary[tw])))
8	("Urinary Incontinence"[Mesh] OR "Nocturia"[Mesh] OR "Urinary Bladder, Overactive"[Mesh] OR “nocturnal diuresis”[tw] OR nocturia[tw] OR (("Urination"[Mesh] OR "Urine"[Mesh] OR urinary[tw] OR urination[tw] OR micturition[tw] OR bladder[tw] OR urine[tw]) AND (incontinence[tw] OR incontinent[tw] OR urgency[tw] OR urge[tw] OR frequency[tw] OR leakage[tw] OR overactive[tw] OR overactivity[tw])))
9	("Uterine Cervical Dysplasia"[Mesh] OR "Papillomavirus Infections"[Mesh] OR "Papillomaviridae"[Mesh] OR “abnormal pap”[tw] OR “abnormal paps”[tw] OR HPV[tw] OR “Human Papilloma Virus”[tw] OR “Human Papilloma Viruses”[tw] OR “Human Papillomavirus”[tw] OR “Papillomavirus Infection”[tw] OR “Papillomavirus Infections”[tw] OR “papillomaviral infection”[tw] OR “papillomaviral infections”[tw] OR ((Cervical[tw] OR cervix[tw] OR vaginal[tw] OR vagina[tw] OR vulvar[tw] OR vulva[tw] OR uterine[tw]) AND (dysplasia[tw] OR dysplasias[tw])))
10	("Menstrual Cycle"[Mesh] OR "Menstruation"[Mesh] OR "Menstruation Disturbances"[Mesh] OR "Menarche"[Mesh] OR menstrual[tw] OR menstruation[tw] OR menstruating[tw] OR menarche[tw] OR menses[tw] OR premenstrual[tw] OR amenorrhea[tw])
11	#2 OR #3 OR #4 OR #5 OR #6 OR #7 OR #8 OR #9 OR #10
12	#1 AND #11

Database: Embase (Elsevier)

Set #	
1	'incarceration'/exp OR 'detention center'/exp OR 'prisoner'/exp OR 'offender'/exp OR 'correctional health care'/exp OR ‘mass incarceration’:ti,ab,kw OR ‘incarcerated women’:ti,ab,kw OR ‘incarcerated people’:ti,ab,kw OR ‘incarcerated persons’:ti,ab,kw OR ‘women incarcerated’:ti,ab,kw OR ‘incarcerated youths’:ti,ab,kw OR ‘incarcerated adolescent’:ti,ab,kw OR ‘incarcerated adolescents’:ti,ab,kw OR ‘adolescents incarcerated’:ti,ab,kw OR ‘incarcerated girl’:ti,ab,kw OR ‘incarcerated girls’:ti,ab,kw OR ‘incarcerated patient’:ti,ab,kw OR ‘incarcerated patients’:ti,ab,kw OR ‘incarcerated individuals’:ti,ab,kw OR ‘incarcerated populations’:ti,ab,kw OR ‘incarcerated population’:ti,ab,kw OR ‘incarcerated adults’:ti,ab,kw OR ‘incarcerated adults’:ti,ab,kw OR ‘incarcerated female’:ti,ab,kw OR ‘incarcerated females’:ti,ab,kw OR carceral:ti,ab,kw OR ‘correctional facility’:ti,ab,kw OR ‘correctional facilities’:ti,ab,kw OR ‘correctional institution’:ti,ab,kw OR ‘correctional institutions’:ti,ab,kw OR ‘correctional center’:ti,ab,kw OR ‘correctional centers’:ti,ab,kw OR ‘correctional centre’:ti,ab,kw OR ‘correctional centres’:ti,ab,kw OR ‘correction facility’:ti,ab,kw OR ‘correction facilities’:ti,ab,kw OR ‘correction institution’:ti,ab,kw OR ‘correction institutions’:ti,ab,kw OR ‘correction center’:ti,ab,kw OR ‘correction centers’:ti,ab,kw OR ‘correction centre’:ti,ab,kw OR ‘correction centres’:ti,ab,kw OR ‘correctional health’:ti,ab,kw OR ‘correctional healthcare’:ti,ab,kw OR ‘correctional care’:ti,ab,kw OR prisoner:ti,ab,kw OR prisoners:ti,ab,kw OR prison:ti,ab,kw OR prisons:ti,ab,kw OR imprisoned:ti,ab,kw OR jail:ti,ab,kw OR jails:ti,ab,kw OR jailed:ti,ab,kw OR inmate:ti,ab,kw OR inmates:ti,ab,kw OR criminal:ti,ab,kw OR criminals:ti,ab,kw OR felon:ti,ab,kw OR felons:ti,ab,kw OR offender:ti,ab,kw OR offenders:ti,ab,kw OR convict:ti,ab,kw OR convicts:ti,ab,kw OR ‘penal institution’:ti,ab,kw OR ‘penal institutions’:ti,ab,kw OR ‘penal facility’:ti,ab,kw OR ‘penal facilities’:ti,ab,kw OR penitentiary:ti,ab,kw OR penitentiaries:ti,ab,kw OR ‘justice involved’:ti,ab,kw OR justice-involved:ti,ab,kw OR ‘detention centers’:ti,ab,kw OR ‘detention center’:ti,ab,kw OR ‘detention centres’:ti,ab,kw OR ‘detention centre’:ti,ab,kw OR ‘detention facility’:ti,ab,kw OR ‘detention facilities’:ti,ab,kw OR ‘juvenile detention’:ti,ab,kw OR ‘youth detention’:ti,ab,kw OR ‘juvenile delinquency’:ti,ab,kw OR ‘juvenile justice’:ti,ab,kw
2	'gynecology'/exp OR 'gynecological examination'/exp OR 'lower urinary tract symptom'/exp OR 'Papanicolaou test'/exp OR 'gynecologic disease'/exp OR gynecology:ti,ab,kw OR gynaecology:ti,ab,kw OR gynecologic:ti,ab,kw OR gynaecologic:ti,ab,kw OR gynecological:ti,ab,kw OR gynaecological:ti,ab,kw OR gynecologist:ti,ab,kw OR gynaecologist:ti,ab,kw OR gynecologists:ti,ab,kw OR gynaecologists:ti,ab,kw OR urogynecologic:ti,ab,kw OR urogynaecologic:ti,ab,kw OR urogynecological:ti,ab,kw OR urogynaecological:ti,ab,kw OR urogynecology:ti,ab,kw OR urogynaecology:ti,ab,kw OR OBGYN:ti,ab,kw OR OB-GYN:ti,ab,kw OR ‘female genital disease’:ti,ab,kw OR ‘female genital diseases’:ti,ab,kw OR ‘adnexal disease’:ti,ab,kw OR ‘adnexal diseases’:ti,ab,kw OR ‘adnexa disease’:ti,ab,kw OR

	‘adnexa diseases’:ti,ab,kw OR ‘vaginal disease’:ti,ab,kw OR ‘vaginal diseases’:ti,ab,kw OR ‘vulvar disease’:ti,ab,kw OR ‘vulvar diseases’:ti,ab,kw OR ‘vulvovaginal disease’:ti,ab,kw OR ‘vulvovaginal diseases’:ti,ab,kw OR ‘ovarian disease’:ti,ab,kw OR ‘ovarian diseases’:ti,ab,kw OR ‘ovarian cysts’:ti,ab,kw OR ‘ovarian cyst’:ti,ab,kw OR ‘Polycystic Ovary Syndrome’:ti,ab,kw OR ‘uterine disease’:ti,ab,kw OR ‘uterine diseases’:ti,ab,kw OR ‘endometrial disease’:ti,ab,kw OR ‘endometrial diseases’:ti,ab,kw OR ‘cervical disease’:ti,ab,kw OR ‘cervical diseases’:ti,ab,kw OR vaginitis:ti,ab,kw OR vulvovaginitis:ti,ab,kw OR vulvitis:ti,ab,kw OR vaginosis:ti,ab,kw OR yeast:ti,ab,kw OR candida:ti,ab,kw OR Candidiasis:ti,ab,kw OR ‘vaginal discharge’:ti,ab,kw OR ‘vaginal discharges’:ti,ab,kw OR ‘vaginal examination’:ti,ab,kw OR ‘vaginal examinations’:ti,ab,kw OR ‘vaginal exam’:ti,ab,kw OR ‘vaginal exams’:ti,ab,kw OR ‘pelvic examination’:ti,ab,kw OR ‘pelvic examinations’:ti,ab,kw OR ‘pelvic exam’:ti,ab,kw OR ‘pelvic exams’:ti,ab,kw OR Papanicolaou:ti,ab,kw OR ‘pap test’:ti,ab,kw OR ‘pap tests’:ti,ab,kw OR ‘pap smear’:ti,ab,kw OR ‘pap smears’:ti,ab,kw OR ‘pap stain’:ti,ab,kw OR ‘pap stains’:ti,ab,kw OR ‘cervical smear’:ti,ab,kw OR ‘cervical smears’:ti,ab,kw OR ‘lower urinary tract symptoms’:ti,ab,kw
3	'uterus bleeding'/exp OR 'vagina bleeding'/exp OR 'menorrhagia and metrorrhagia'/exp OR ‘uterine hemorrhage’:ti,ab,kw OR ‘uterine hemorrhages’:ti,ab,kw OR ‘uterine hemorrhaging’:ti,ab,kw OR ‘uterus hemorrhage’:ti,ab,kw OR ‘uterus hemorrhages’:ti,ab,kw OR ‘uterus hemorrhaging’:ti,ab,kw OR ‘uterine haemorrhage’:ti,ab,kw OR ‘uterine haemorrhages’:ti,ab,kw OR ‘uterine haemorrhaging’:ti,ab,kw OR ‘uterus haemorrhage’:ti,ab,kw OR ‘uterus haemorrhages’:ti,ab,kw OR ‘uterus haemorrhaging’:ti,ab,kw OR ‘uterine bleeding’:ti,ab,kw OR ‘uterus bleeding’:ti,ab,kw OR ‘vaginal hemorrhage’:ti,ab,kw OR ‘vaginal hemorrhages’:ti,ab,kw OR ‘vaginal hemorrhaging’:ti,ab,kw OR ‘vaginal haemorrhage’:ti,ab,kw OR ‘vaginal haemorrhages’:ti,ab,kw OR ‘vaginal haemorrhaging’:ti,ab,kw OR ‘vaginal bleeding’:ti,ab,kw OR ‘vagina hemorrhage’:ti,ab,kw OR ‘vagina hemorrhages’:ti,ab,kw OR ‘vagina hemorrhaging’:ti,ab,kw OR ‘vagina haemorrhage’:ti,ab,kw OR ‘vagina haemorrhages’:ti,ab,kw OR ‘vagina haemorrhaging’:ti,ab,kw OR ‘vagina bleeding’:ti,ab,kw OR ‘abnormal bleeding’:ti,ab,kw OR ‘irregular bleeding’:ti,ab,kw OR ‘abnormal menstrual bleeding’:ti,ab,kw OR ‘abnormal menses’:ti,ab,kw OR ‘Intermenstrual Bleeding’:ti,ab,kw OR ‘Dysfunctional Uterine Bleeding’:ti,ab,kw OR ‘Breakthrough Bleeding’:ti,ab,kw OR ‘Bleeding Between Periods’:ti,ab,kw OR ‘bleeding between menses’:ti,ab,kw OR ‘heavy menstrual bleeding’:ti,ab,kw OR ‘heavy menstruation’:ti,ab,kw OR ‘heavy period’:ti,ab,kw OR ‘heavy periods’:ti,ab,kw OR ‘heavy menses’:ti,ab,kw OR hypermenorrhea:ti,ab,kw OR hypermenorrhoea:ti,ab,kw OR menorrhagia:ti,ab,kw OR metrorrhagia:ti,ab,kw OR menometrorrhagia:ti,ab,kw OR ‘irregular menstrual bleeding’:ti,ab,kw OR ‘irregular menses’:ti,ab,kw

4	'menopause'/exp OR 'menopause related disorder'/exp OR menopaus*:ti,ab,kw OR premenopaus*:ti,ab,kw OR postmenopaus*:ti,ab,kw OR perimenopause*:ti,ab,kw OR ‘hot flashes’:ti,ab,kw OR ‘hot flushes’:ti,ab,kw
5	'pelvic pain'/exp OR dysmenorrhea:ti,ab,kw OR dysmenorrheas:ti,ab,kw OR ‘piriformis muscle syndrome’:ti,ab,kw OR ‘piriformis syndrome’:ti,ab,kw OR endometriosis:ti,ab,kw OR endometrioses:ti,ab,kw OR (('pain'/exp OR pain*:ti,ab,kw) AND ('pelvis'/exp OR pelvis:ti,ab,kw OR pelvic:ti,ab,kw))
6	'female genital tract tumor'/exp OR ‘Squamous Intraepithelial Lesions of the Cervix’:ti,ab,kw OR (('female genital system'/exp OR ‘female genital’:ti,ab,kw OR ‘female genitals’:ti,ab,kw OR ‘female genitalia’:ti,ab,kw OR ‘female reproductive’:ti,ab,kw OR Gynecologic:ti,ab,kw OR cervical:ti,ab,kw OR cervix:ti,ab,kw OR endometrium:ti,ab,kw OR endometria:ti,ab,kw OR endometrial:ti,ab,kw OR ovary:ti,ab,kw OR ovaries:ti,ab,kw OR ovarian:ti,ab,kw OR uterus:ti,ab,kw OR uteri:ti,ab,kw OR womb:ti,ab,kw OR wombs:ti,ab,kw OR uterine:ti,ab,kw OR vagina:ti,ab,kw OR vaginas:ti,ab,kw OR vaginal:ti,ab,kw OR vulva:ti,ab,kw OR vulvas:ti,ab,kw OR vulvar:ti,ab,kw OR ‘fallopian tube’:ti,ab,kw OR ‘fallopian tubes’:ti,ab,kw) AND ('neoplasm'/exp OR neoplasm:ti,ab,kw OR neoplasms:ti,ab,kw OR neoplasia:ti,ab,kw OR cancer:ti,ab,kw OR cancers:ti,ab,kw OR cancerous:ti,ab,kw OR carcinoma:ti,ab,kw OR carcinomas:ti,ab,kw OR malignant:ti,ab,kw OR malignancy:ti,ab,kw OR malignancies:ti,ab,kw OR metastasis:ti,ab,kw OR metastases:ti,ab,kw OR metastatic:ti,ab,kw OR tumor:ti,ab,kw OR tumors:ti,ab,kw OR tumour:ti,ab,kw OR tumours:ti,ab,kw OR sarcoma:ti,ab,kw OR sarcomas:ti,ab,kw OR oncology:ti,ab,kw OR oncologist:ti,ab,kw OR oncologists:ti,ab,kw OR oncologica:ti,ab,kw))
7	('pelvic organ prolapse'/exp OR (('prolapse'/exp OR prolapse:ti,ab,kw OR prolapses:ti,ab,kw) AND ('pelvis'/exp OR 'vagina'/exp OR 'uterus'/exp OR pelvis:ti,ab,kw OR pelvic:ti,ab,kw OR uterine:ti,ab,kw OR visceral:ti,ab,kw OR rectal:ti,ab,kw OR vaginal:ti,ab,kw OR vaginal:ti,ab,kw OR urogenital:ti,ab,kw OR genital:ti,ab,kw OR urinary:ti,ab,kw OR genitourinary:ti,ab,kw)))
8	('urine incontinence'/exp OR 'nocturia'/exp OR 'overactive bladder'/exp OR ‘nocturnal diuresis’:ti,ab,kw OR nocturia:ti,ab,kw OR (('micturition'/exp OR 'urine'/exp OR urinary:ti,ab,kw OR urination:ti,ab,kw OR micturition:ti,ab,kw OR bladder:ti,ab,kw OR urine:ti,ab,kw) AND (incontinence:ti,ab,kw OR incontinent:ti,ab,kw OR urgency:ti,ab,kw OR urge:ti,ab,kw OR frequency:ti,ab,kw OR leakage:ti,ab,kw OR overactive:ti,ab,kw OR overactivity:ti,ab,kw)))
9	('uterine cervix dysplasia'/exp OR 'papillomavirus infection'/exp OR 'Papillomaviridae'/exp OR ‘abnormal pap’:ti,ab,kw OR ‘abnormal paps’:ti,ab,kw OR HPV:ti,ab,kw OR ‘Human Papilloma Virus’:ti,ab,kw OR ‘Human Papilloma Viruses’:ti,ab,kw OR ‘Human Papillomavirus’:ti,ab,kw OR ‘Papillomavirus Infection’:ti,ab,kw OR ‘Papillomavirus Infections’:ti,ab,kw OR ‘papillomaviral

	infection’:ti,ab,kw OR ‘papillomaviral infections’:ti,ab,kw OR ((Cervical:ti,ab,kw OR cervix:ti,ab,kw OR vaginal:ti,ab,kw OR vagina:ti,ab,kw OR vulvar:ti,ab,kw OR vulva:ti,ab,kw OR uterine:ti,ab,kw) AND ('dysplasia'/exp OR dysplasia:ti,ab,kw OR dysplasias:ti,ab,kw)))
10	('menstrual cycle'/exp OR 'menstruation'/exp OR 'menstruation disorder'/exp OR 'menarche'/exp OR menstrual:ti,ab,kw OR menstruation:ti,ab,kw OR menstruating:ti,ab,kw OR menarche:ti,ab,kw OR menses:ti,ab,kw OR premenstrual:ti,ab,kw OR amenorrhea:ti,ab,kw)
11	#2 OR #3 OR #4 OR #5 OR #6 OR #7 OR #8 OR #9 OR #10
12	#1 AND #11
13	#12 AND ('article'/it OR 'article in press'/it OR 'conference paper'/it OR 'conference review'/it OR 'editorial'/it OR 'erratum'/it OR 'letter'/it OR 'note'/it OR 'review'/it)

Scopus

Set #	
1	TITLE-ABS ("mass incarceration" OR "incarcerated women" OR "incarcerated people" OR "incarcerated persons" OR "women incarcerated" OR "incarcerated youths" OR "incarcerated adolescent" OR "incarcerated adolescents" OR "adolescents incarcerated" OR "incarcerated girl" OR "incarcerated girls" OR "incarcerated patient" OR "incarcerated patients" OR "incarcerated individuals" OR "incarcerated populations" OR "incarcerated population" OR "incarcerated adults" OR "incarcerated adults" OR "incarcerated female" OR "incarcerated females" OR carceral OR "correctional facility" OR "correctional facilities" OR "correctional institution" OR "correctional institutions" OR "correctional center" OR "correctional centers" OR "correctional centre" OR "correctional centres" OR "correction facility" OR "correction facilities" OR "correction institution" OR "correction institutions" OR "correction center" OR "correction centers" OR "correction centre" OR "correction centres" OR "correctional health" OR "correctional healthcare" OR "correctional care" OR prisoner OR prisoners OR prison OR prisons OR imprisoned OR jail OR jails OR jailed OR inmate OR inmates OR criminal OR criminals OR felon OR felons OR offender OR offenders OR convict OR convicts OR "penal institution" OR "penal institutions" OR "penal facility" OR "penal facilities" OR penitentiary OR penitentiaries OR "justice involved" OR justice-involved OR "detention centers" OR "detention center" OR "detention centres" OR "detention centre" OR "detention facility" OR "detention facilities" OR "juvenile detention" OR "youth detention" OR "juvenile delinquency" OR "juvenile justice")

2	TITLE-ABS (gynecology OR gynaecology OR gynecologic OR gynaecologic OR gynecological OR gynaecological OR gynecologist OR gynaecologist OR gynecologists OR gynaecologists OR urogynecologic OR urogynaecologic OR urogynecological OR urogynaecological OR urogynecology OR urogynaecology OR obgyn OR ob-gyn OR "female genital disease" OR "female genital diseases" OR "adnexal disease" OR "adnexal diseases" OR "adnexa disease" OR "adnexa diseases" OR "vaginal disease" OR "vaginal diseases" OR "vulvar disease" OR "vulvar diseases" OR "vulvovaginal disease" OR "vulvovaginal diseases" OR "vulvar diseases" OR "ovarian disease" OR "ovarian diseases" OR "ovarian cysts" OR "ovarian cyst" OR "Polycystic Ovary Syndrome" OR "uterine disease" OR "uterine diseases" OR "endometrial disease" OR "endometrial diseases" OR "cervical disease" OR "cervical diseases" OR vaginitis OR vulvovaginitis OR vulvitis OR vaginosis OR yeast OR candida OR candidiasis OR "vaginal discharge" OR "vaginal discharges" OR "vaginal examination" OR "vaginal examinations" OR "vaginal exam" OR "vaginal exams" OR "pelvic examination" OR "pelvic examinations" OR "pelvic exam" OR "pelvic exams" OR papanicolaou OR "pap test" OR "pap tests" OR "pap smear" OR "pap smears" OR "pap stain" OR "pap stains" OR "cervical smear" OR "cervical smears" OR "lower urinary tract symptoms")
3	TITLE-ABS ("uterine hemorrhage" OR "uterine hemorrhages" OR "uterine hemorrhaging" OR "uterus hemorrhage" OR "uterus hemorrhages" OR "uterus hemorrhaging" OR "uterine haemorrhage" OR "uterine haemorrhages" OR "uterine haemorrhaging" OR "uterus haemorrhage" OR "uterus haemorrhages" OR "uterus haemorrhaging" OR "uterine bleeding" OR "uterus bleeding" OR "vaginal hemorrhage" OR "vaginal hemorrhages" OR "vaginal hemorrhaging" OR "vaginal haemorrhage" OR "vaginal haemorrhages" OR "vaginal haemorrhaging" OR "vaginal bleeding" OR "vagina hemorrhage" OR "vagina hemorrhages" OR "vagina hemorrhaging" OR "vagina haemorrhage" OR "vagina haemorrhages" OR "vagina haemorrhaging" OR "vagina bleeding" OR "abnormal bleeding" OR "irregular bleeding" OR "abnormal menstrual bleeding" OR "abnormal menses" OR "Intermenstrual Bleeding" OR "Dysfunctional Uterine Bleeding" OR "Breakthrough Bleeding" OR "Bleeding Between Periods" OR "bleeding between menses" OR "heavy menstrual bleeding" OR "heavy menstruation" OR "heavy period" OR "heavy periods" OR "heavy menses" OR hypermenorrhea OR hypermenorrhoea OR menorrhagia OR metrorrhagia OR menometrorrhagia OR "irregular menstrual bleeding" OR "irregular menses")
4	TITLE-ABS (menopaus* OR premenopaus* OR postmenopaus* OR perimenopause* OR "hot flashes" OR "hot flushes")
5	TITLE-ABS (dysmenorrhea OR dysmenorrheas OR "piriformis muscle syndrome" OR "piriformis syndrome" OR endometriosis OR endometrioses OR ((pain*) AND (pelvis OR pelvic)))

6	TITLE-ABS ("Squamous Intraepithelial Lesions of the Cervix" OR (("female genital" OR "female genitals" OR "female genitalia" OR "female reproductive" OR gynecologic OR cervical OR cervix OR endometrium OR endometria OR endometrial OR ovary OR ovaries OR ovarian OR uterus OR uteri OR womb OR wombs OR uterine OR vagina OR vaginas OR vaginal OR vulva OR vulvas OR vulvar OR "fallopian tube" OR "fallopian tubes") AND (neoplasm OR neoplasms OR neoplasia OR cancer OR cancers OR cancerous OR carcinoma OR carcinomas OR malignant OR malignancy OR malignancies OR metastasis OR metastases OR metastatic OR tumor OR tumors OR tumour OR tumours OR sarcoma OR sarcomas OR oncology OR oncologist OR oncologists OR oncologica)))
7	TITLE-ABS ((prolapse OR prolapses) AND (pelvis OR pelvic OR uterine OR visceral OR rectal OR vaginal OR vaginal OR urogenital OR genital OR urinary OR genitourinary))
8	TITLE-ABS ("nocturnal diuresis" OR nocturia OR ((urinary OR urination OR micturition OR bladder OR urine) AND (incontinence OR incontinent OR urgency OR urge OR frequency OR leakage OR overactive OR overactivity)))
9	TITLE-ABS ("abnormal pap" OR "abnormal paps" OR hpv OR "Human Papilloma Virus" OR "Human Papilloma Viruses" OR "Human Papillomavirus" OR "Papillomavirus Infection" OR "Papillomavirus Infections" OR "papillomaviral infection" OR "papillomaviral infections" OR ((cervical OR cervix OR vaginal OR vagina OR vulvar OR vulva OR uterine) AND (dysplasia OR dysplasias)))
10	TITLE-ABS (menstrual OR menstruation OR menstruating OR menarche OR menses OR premenstrual OR amenorrhea)
11	#2 OR #3 OR #4 OR #5 OR #6 OR #7 OR #8 OR #9 OR #10
12	#1 AND #11

Database: APA PsycInfo (EBSCO*host*)

Set #	
1	DE "Incarceration" OR DE "Correctional Institutions" OR DE "Prisons" OR DE "Reformatories" OR DE "Prisoners" OR DE "Prisoners of War" OR DE "Criminal Offenders" OR DE "Female Criminal Offenders" OR DE "Male Criminal Offenders" OR DE "Mentally Ill Offenders" OR DE "Juvenile Delinquency" OR DE "Female Delinquency" OR DE "Male Delinquency" OR DE "Predelinquent Youth" OR DE "Juvenile Justice" OR “mass incarceration” OR "incarcerated women" OR "incarcerated people" OR "incarcerated persons" OR "women incarcerated" OR "incarcerated youths"

	OR "incarcerated adolescent" OR "incarcerated adolescents" OR “adolescents incarcerated” OR "incarcerated girl" OR "incarcerated girls" OR "incarcerated patient" OR "incarcerated patients" OR “incarcerated individuals” OR “incarcerated populations” OR “incarcerated population” OR “incarcerated adults” OR “incarcerated adults” OR “incarcerated female” OR “incarcerated females” OR carceral OR "correctional facility" OR "correctional facilities" OR "correctional institution" OR "correctional institutions" OR "correctional center" OR "correctional centers" OR "correctional centre" OR "correctional centres" OR "correction facility" OR "correction facilities" OR "correction institution" OR "correction institutions" OR "correction center" OR "correction centers" OR "correction centre" OR "correction centres" OR “correctional health” OR “correctional healthcare” OR “correctional care” OR prisoner OR prisoners OR prison OR prisons OR imprisoned OR jail OR jails OR jailed OR inmate OR inmates OR criminal OR criminals OR felon OR felons OR offender OR offenders OR convict OR convicts OR "penal institution" OR "penal institutions" OR "penal facility" OR "penal facilities" OR penitentiary OR penitentiaries OR “justice involved” OR justice-involved OR “detention centers” OR “detention center” OR “detention centres” OR “detention centre” OR “detention facility” OR “detention facilities” OR “juvenile detention” OR “youth detention” OR “juvenile delinquency” OR “juvenile justice”
2	DE "Gynecology" OR DE "Gynecological Disorders" OR gynecology OR gynaecology OR gynecologic OR gynaecologic OR gynecological OR gynaecological OR gynecologist OR gynaecologist OR gynecologists OR gynaecologists OR urogynecologic OR urogynaecologic OR urogynecological OR urogynaecological OR urogynecology OR urogynaecology OR OBGYN OR OB-GYN OR “female genital disease” OR “female genital diseases” OR “adnexal disease” OR “adnexal diseases” OR “adnexa disease” OR “adnexa diseases” OR “vaginal disease” OR “vaginal diseases” OR “vulvar disease” OR “vulvar diseases” OR “vulvovaginal disease” OR “vulvovaginal diseases” OR “vulvar diseases” OR “ovarian disease” OR “ovarian diseases” OR “ovarian cysts” OR “ovarian cyst” OR “Polycystic Ovary Syndrome” OR “uterine disease” OR “uterine diseases” OR “endometrial disease” OR “endometrial diseases” OR “cervical disease” OR “cervical diseases” OR vaginitis OR vulvovaginitis OR vulvitis OR vaginosis OR yeast OR candida OR Candidiasis OR “vaginal discharge” OR “vaginal discharges” OR “vaginal examination” OR “vaginal examinations” OR “vaginal exam” OR “vaginal exams” OR “pelvic examination” OR “pelvic examinations” OR “pelvic exam” OR “pelvic exams” OR Papanicolaou OR “pap test” OR “pap tests” OR “pap smear” OR “pap smears” OR “pap stain” OR “pap stains” OR “cervical smear” OR “cervical smears” OR “lower urinary tract symptoms”
3	"uterine hemorrhage" OR "uterine hemorrhages" OR "uterine hemorrhaging" OR "uterus hemorrhage" OR "uterus hemorrhages" OR "uterus hemorrhaging" OR "uterine haemorrhage" OR "uterine haemorrhages" OR "uterine haemorrhaging" OR "uterus haemorrhage" OR "uterus haemorrhages" OR "uterus haemorrhaging" OR “uterine bleeding” OR “uterus bleeding” OR "vaginal hemorrhage" OR "vaginal hemorrhages"

	OR "vaginal hemorrhaging" OR "vaginal haemorrhage" OR "vaginal haemorrhages" OR "vaginal haemorrhaging" OR “vaginal bleeding” OR "vagina hemorrhage" OR "vagina hemorrhages" OR "vagina hemorrhaging" OR "vagina haemorrhage" OR "vagina haemorrhages" OR "vagina haemorrhaging" OR “vagina bleeding” OR “abnormal bleeding” OR “irregular bleeding” OR “abnormal menstrual bleeding” OR “abnormal menses” OR “Intermenstrual Bleeding” OR “Dysfunctional Uterine Bleeding” OR “Breakthrough Bleeding” OR “Bleeding Between Periods” OR “bleeding between menses” OR “heavy menstrual bleeding” OR “heavy menstruation” OR “heavy period” OR “heavy periods” OR “heavy menses” OR hypermenorrhea OR hypermenorrhoea OR menorrhagia OR metrorrhagia OR menometrorrhagia OR “irregular menstrual bleeding” OR “irregular menses”
4	DE "Menopause" OR menopaus* OR premenopaus* OR postmenopaus* OR perimenopause* OR “hot flashes” OR “hot flushes”
5	dysmenorrhea OR dysmenorrheas OR “piriformis muscle syndrome” OR “piriformis syndrome” OR endometriosis OR endometrioses OR ((DE "Pain" OR DE "Acute Pain" OR DE "Aphagia" OR DE "Chronic Pain" OR DE "Pain Management" OR pain*) AND (pelvis OR pelvic))
6	“Squamous Intraepithelial Lesions of the Cervix” OR ((DE "Female Genitalia" OR DE "Ovaries" OR DE "Uterus" OR DE "Vagina" OR “female genital” OR “female genitals” OR “female genitalia” OR “female reproductive” OR Gynecologic OR cervical OR cervix OR endometrium OR endometria OR endometrial OR ovary OR ovaries OR ovarian OR uterus OR uteri OR womb OR wombs OR uterine OR vagina OR vaginas OR vaginal OR vulva OR vulvas OR vulvar OR “fallopian tube” OR “fallopian tubes”) AND (DE "Neoplasms" OR DE "Benign Neoplasms" OR DE "Metastasis" OR DE "Terminal Cancer" OR neoplasm OR neoplasms OR neoplasia OR cancer OR cancers OR cancerous OR carcinoma OR carcinomas OR malignant OR malignancy OR malignancies OR metastasis OR metastases OR metastatic OR tumor OR tumors OR tumour OR tumours OR sarcoma OR sarcomas OR oncology OR oncologist OR oncologists OR oncologica))
7	((prolapse OR prolapses) AND (DE "Vagina" OR DE "Uterus" OR pelvis OR pelvic OR uterine OR visceral OR rectal OR vaginal OR vaginal OR urogenital OR genital OR urinary OR genitourinary))
8	(DE "Urinary Incontinence" OR “nocturnal diuresis” OR nocturia OR ((DE "Urination" OR DE "Diuresis" OR DE "Urine" OR urinary OR urination OR micturition OR bladder OR urine) AND (incontinence OR incontinent OR urgency OR urge OR frequency OR leakage OR overactive OR overactivity)))
9	(DE "Human Papillomavirus" OR “abnormal pap” OR “abnormal paps” OR HPV OR “Human Papilloma Virus” OR “Human Papilloma Viruses” OR “Human Papillomavirus” OR “Papillomavirus Infection” OR “Papillomavirus Infections” OR

	“papillomaviral infection” OR “papillomaviral infections” OR ((Cervical OR cervix OR vaginal OR vagina OR vulvar OR vulva OR uterine) AND (dysplasia OR dysplasias)))
10	(DE "Menstrual Cycle" OR DE "Menstruation" OR DE "Ovulation" OR DE "Menstrual Disorders" OR DE "Amenorrhea" OR DE "Dysmenorrhea" OR DE "Premenstrual Dysphoric Disorder" OR DE "Menarche" OR menstrual OR menstruation OR menstruating OR menarche OR menses OR premenstrual OR amenorrhea)
11	#2 OR #3 OR #4 OR #5 OR #6 OR #7 OR #8 OR #9 OR #10
12	#1 AND #11

Database: CINAHL Plus with Full Text (EBSCO*host*)

Set #	
1	MH "Correctional Facilities" OR MH "Prisoners" OR MH "Public Offenders+" OR MH "Juvenile Delinquency" OR MH "Correctional Health Services" OR MH "Correctional Health Nursing" OR “mass incarceration” OR "incarcerated women" OR "incarcerated people" OR "incarcerated persons" OR "women incarcerated" OR "incarcerated youths" OR "incarcerated adolescent" OR "incarcerated adolescents" OR “adolescents incarcerated” OR "incarcerated girl" OR "incarcerated girls" OR "incarcerated patient" OR "incarcerated patients" OR “incarcerated individuals” OR “incarcerated populations” OR “incarcerated population” OR “incarcerated adults” OR “incarcerated adults” OR “incarcerated female” OR “incarcerated females” OR carceral OR "correctional facility" OR "correctional facilities" OR "correctional institution" OR "correctional institutions" OR "correctional center" OR "correctional centers" OR "correctional centre" OR "correctional centres" OR "correction facility" OR "correction facilities" OR "correction institution" OR "correction institutions" OR "correction center" OR "correction centers" OR "correction centre" OR "correction centres" OR “correctional health” OR “correctional healthcare” OR “correctional care” OR prisoner OR prisoners OR prison OR prisons OR imprisoned OR jail OR jails OR jailed OR inmate OR inmates OR criminal OR criminals OR felon OR felons OR offender OR offenders OR convict OR convicts OR "penal institution" OR "penal institutions" OR "penal facility" OR "penal facilities" OR penitentiary OR penitentiaries OR “justice involved” OR justice-involved OR “detention centers” OR “detention center” OR “detention centres” OR “detention centre” OR “detention facility” OR “detention facilities” OR “juvenile detention” OR “youth detention” OR “juvenile delinquency” OR “juvenile justice”
2	MH "Gynecology" OR MH "Genital Diseases, Female" OR MH "Adnexal Diseases" OR MH "Gynatresia" OR MH "Herlyn-Werner-Wunderlich Syndrome" OR MH

	"Uterine Diseases+" OR MH "Endometrial Diseases+" OR MH "Hematometra" OR MH "Pyometra" OR MH "Uterine Hemorrhage+" OR MH "Uterine Inversion" OR MH "Vaginal Diseases+" OR MH "Vulvar Diseases+" OR MH "Cervical Smears+" OR gynecology OR gynaecology OR gynecologic OR gynaecologic OR gynecological OR gynaecological OR gynecologist OR gynaecologist OR gynecologists OR gynaecologists OR urogynecologic OR urogynaecologic OR urogynecological OR urogynaecological OR urogynecology OR urogynaecology OR OBGYN OR OB-GYN OR “female genital disease” OR “female genital diseases” OR “adnexal disease” OR “adnexal diseases” OR “adnexa disease” OR “adnexa diseases” OR “vaginal disease” OR “vaginal diseases” OR “vulvar disease” OR “vulvar diseases” OR “vulvovaginal disease” OR “vulvovaginal diseases” OR “vulvar diseases” OR “ovarian disease” OR “ovarian diseases” OR “ovarian cysts” OR “ovarian cyst” OR “Polycystic Ovary Syndrome” OR “uterine disease” OR “uterine diseases” OR “endometrial disease” OR “endometrial diseases” OR “cervical disease” OR “cervical diseases” OR vaginitis OR vulvovaginitis OR vulvitis OR vaginosis OR yeast OR candida OR Candidiasis OR “vaginal discharge” OR “vaginal discharges” OR “vaginal examination” OR “vaginal examinations” OR “vaginal exam” OR “vaginal exams” OR “pelvic examination” OR “pelvic examinations” OR “pelvic exam” OR “pelvic exams” OR Papanicolaou OR “pap test” OR “pap tests” OR “pap smear” OR “pap smears” OR “pap stain” OR “pap stains” OR “cervical smear” OR “cervical smears” OR “lower urinary tract symptoms”
3	MH "Uterine Hemorrhage" OR MH "Metrorrhagia" OR MH "Menorrhagia" OR "uterine hemorrhage" OR "uterine hemorrhages" OR "uterine hemorrhaging" OR "uterus hemorrhage" OR "uterus hemorrhages" OR "uterus hemorrhaging" OR "uterine haemorrhage" OR "uterine haemorrhages" OR "uterine haemorrhaging" OR "uterus haemorrhage" OR "uterus haemorrhages" OR "uterus haemorrhaging" OR “uterine bleeding” OR “uterus bleeding” OR "vaginal hemorrhage" OR "vaginal hemorrhages" OR "vaginal hemorrhaging" OR "vaginal haemorrhage" OR "vaginal haemorrhages" OR "vaginal haemorrhaging" OR “vaginal bleeding” OR "vagina hemorrhage" OR "vagina hemorrhages" OR "vagina hemorrhaging" OR "vagina haemorrhage" OR "vagina haemorrhages" OR "vagina haemorrhaging" OR “vagina bleeding” OR “abnormal bleeding” OR “irregular bleeding” OR “abnormal menstrual bleeding” OR “abnormal menses” OR “Intermenstrual Bleeding” OR “Dysfunctional Uterine Bleeding” OR “Breakthrough Bleeding” OR “Bleeding Between Periods” OR “bleeding between menses” OR “heavy menstrual bleeding” OR “heavy menstruation” OR “heavy period” OR “heavy periods” OR “heavy menses” OR hypermenorrhea OR hypermenorrhoea OR menorrhagia OR metrorrhagia OR menometrorrhagia OR “irregular menstrual bleeding” OR “irregular menses”
4	MH "Menopause+" OR menopaus* OR premenopaus* OR postmenopaus* OR perimenopause* OR “hot flashes” OR “hot flushes”
5	MH "Pelvic Pain+" OR dysmenorrhea OR dysmenorrheas OR “piriformis muscle syndrome” OR “piriformis syndrome” OR endometriosis OR endometrioses OR ((MH

	"Pain+" OR MH "Pain Management" OR pain*) AND (MH "Pelvis+" OR pelvis OR pelvic))
6	MH "Genital Neoplasms, Female+" OR "Squamous Intraepithelial Lesions of the Cervix" OR ((MH "Genitalia, Female+" OR "female genital" OR "female genitals" OR "female genitalia" OR "female reproductive" OR Gynecologic OR cervical OR cervix OR endometrium OR endometria OR endometrial OR ovary OR ovaries OR ovarian OR uterus OR uteri OR womb OR wombs OR uterine OR vagina OR vaginas OR vaginal OR vulva OR vulvas OR vulvar OR "fallopian tube" OR "fallopian tubes") AND (MH "Neoplasms+" OR neoplasm OR neoplasms OR neoplasia OR cancer OR cancers OR cancerous OR carcinoma OR carcinomas OR malignant OR malignancy OR malignancies OR metastasis OR metastases OR metastatic OR tumor OR tumors OR tumour OR tumours OR sarcoma OR sarcomas OR oncology OR oncologist OR oncologists OR oncologica))
7	(MH "Pelvic Organ Prolapse+" OR ((MH "Prolapse" OR prolapse OR prolapses) AND (MH "Pelvis+" OR MH "Vagina" OR MH "Uterus" OR pelvis OR pelvic OR uterine OR visceral OR rectal OR vaginal OR vaginal OR urogenital OR genital OR urinary OR genitourinary)))
8	(MH "Urinary Incontinence+" OR MH "Overactive Bladder" OR "nocturnal diuresis" OR nocturia OR ((MH "Urination" OR MH "Urine" OR urinary OR urination OR micturition OR bladder OR urine) AND (incontinence OR incontinent OR urgency OR urge OR frequency OR leakage OR overactive OR overactivity)))
9	(MH "Cervix Dysplasia" OR MH "Papillomavirus Infections+ OR MH "Papillomaviruses" OR "abnormal pap" OR "abnormal paps" OR HPV OR "Human Papilloma Virus" OR "Human Papilloma Viruses" OR "Human Papillomavirus" OR "Papillomavirus Infection" OR "Papillomavirus Infections" OR "papillomaviral infection" OR "papillomaviral infections" OR ((Cervical OR cervix OR vaginal OR vagina OR vulvar OR vulva OR uterine) AND (dysplasia OR dysplasias)))
10	(MH "Menstrual Cycle+" OR MH "Menstrual and Perimenopausal Disorders+" OR MH "Menstruation" OR MH "Menarche" OR menstrual OR menstruation OR menstruating OR menarche OR menses OR premenstrual OR amenorrhea)
11	#2 OR #3 OR #4 OR #5 OR #6 OR #7 OR #8 OR #9 OR #10
12	#1 AND #11

Registry: ClinicalTrials.gov (Advanced search platform)
Searched in Condition or disease field AND Other terms field

Set #	

1	(Adnexal Diseases OR Atypical Squamous Cells of the Cervix OR Candidiasis, Vulvovaginal OR Carcinoma, Endometrioid OR Carcinoma, Ovarian Epithelial OR Endometrial Neoplasms OR Endometriosis OR Fallopian Tube Diseases OR Fallopian Tube Neoplasms) AND (incarceration OR incarcerated OR jail OR prison OR offender OR "correctional facilities" OR inmates)
2	(Genital Neoplasms, Female OR Menopause, Premature OR Menorrhagia OR Metrorrhagia OR Ovarian Cysts OR Ovarian Diseases OR Ovarian Neoplasms OR Pelvic Floor Disorders OR Polycystic Ovary Syndrome OR Polyuria) AND (incarceration OR incarcerated OR jail OR prison OR offender OR "correctional facilities" OR inmates)
3	(Squamous Intraepithelial Lesions of the Cervix OR Urinary Bladder, Overactive OR Urinary Incontinence OR Uterine Cervical Diseases OR Uterine Cervical Dysplasia OR Uterine Cervical Neoplasms) AND (incarceration OR incarcerated OR jail OR prison OR offender OR "correctional facilities" OR inmates)
4	(Uterine Diseases OR Uterine Hemorrhage OR Uterine Neoplasms OR Uterine Prolapse OR Vaginal Discharge OR Vaginal Diseases OR Vaginal Neoplasms OR Vaginitis) AND (incarceration OR incarcerated OR jail OR prison OR offender OR "correctional facilities" OR inmates)
5	(Vaginosis, Bacterial OR Vulvar Diseases OR Vulvar Neoplasms OR Vulvovaginitis) AND (incarceration OR incarcerated OR jail OR prison OR offender OR "correctional facilities" OR inmates)
6	#1 OR #2 OR #3 OR #4 OR #5
